# An automatically curated first-principles database of ferroelectrics

**DOI:** 10.1038/s41597-020-0407-9

**Published:** 2020-03-03

**Authors:** Tess E. Smidt, Stephanie A. Mack, Sebastian E. Reyes-Lillo, Anubhav Jain, Jeffrey B. Neaton

**Affiliations:** 1 Department of Physics, University of California, Berkeley, California 94720 United States; 2 Molecular Foundry, Lawrence Berkeley National Laboratory, Berkeley, California 94720 United States; 3 Materials Sciences Division, Lawrence Berkeley National Laboratory, Berkeley, California 94720 United States; 4 Departamento de Ciencias Físicas, Universidad Andres Bello, Santiago, 837-0136 Chile; 5 Kavli Energy Nanosciences Institute at Berkeley, Berkeley, California 94720 United States

**Keywords:** Electronic structure, Ferroelectrics and multiferroics

## Abstract

Ferroelectric materials have technological applications in information storage and electronic devices. The ferroelectric polar phase can be controlled with external fields, chemical substitution and size-effects in bulk and ultrathin film form, providing a platform for future technologies and for exploratory research. In this work, we integrate spin-polarized density functional theory (DFT) calculations, crystal structure databases, symmetry tools, workflow software, and a custom analysis toolkit to build a library of known, previously-proposed, and newly-proposed ferroelectric materials. With our automated workflow, we screen over 67,000 candidate materials from the Materials Project database to generate a dataset of 255 ferroelectric candidates, and propose 126 new ferroelectric materials. We benchmark our results against experimental data and previous first-principles results. The data provided includes atomic structures, output files, and DFT values of band gaps, energies, and the spontaneous polarization for each ferroelectric candidate. We contribute our workflow and analysis code to the open-source python packages atomate and pymatgen so others can conduct analogous symmetry driven searches for ferroelectrics and related phenomena.

## Abstract (summary)

**Table:** 

Measurement(s)	ferroelectrics • Workflow
Technology Type(s)	digital curation

Machine-accessible metadata file describing the reported data: 10.6084/m9.figshare.11702073

## Background & Summary

High-throughput screening of material databases integrated with first-principles calculations has been increasingly successful in the discovery of new functional materials^1–4^. While many of the individual components for performing high-throughput searches exist, the infrastructure needed to connect and automate all the necessary components is still under development. The identification of ferroelectrics through symmetry arguments has been an active area of research^5–16^. Moreover, lists of known ferroelectrics have been previously curated^17–23^. However, the identification of new ferroelectrics has yet to be automated in a manner readily applicable to emerging materials databases^24–30^. Automated high-throughput searches for ferroelectric candidates would provide a valuable guide for in-depth computational studies and experimental efforts.

Ferroelectrics have important technological applications, such as in tunable capacitors, non-volatile random access memory devices, and electro-optical data storage. In addition, ferroelectrics are capable of displaying couplings between their electronic degrees of freedom with magnetic or lattice degrees of freedom in multiferroic materials. Ferroelectricity often arises from a structural phase transition between a high-symmetry nonpolar structural phase to a low-symmetry polar structural phase with decreasing temperature, resulting in the emergence of a spontaneous polarization^31–33^. In this scenario, the atomic geometry of the nonpolar structure can continuously distort such that the new polar structure has a subset of the symmetries of the original structure, satisfying the requirements of a second-order phase transition; in these cases, the polar space group must be an isotropy subgroup of the nonpolar space group, which is a stronger requirement than they only share a simple group-subgroup relation^34–36^.

Thus, certain ferroelectrics can be systematically screened by searching for pairs of nonpolar and polar structures related by a small symmetry-breaking distortion. In the late 1980s, Abrahams performed some of the earliest searches for ferroelectrics in crystallographic databases using symmetry criteria^5,6^. More recently, automated searches for new ferroelectric candidates have used symmetry arguments to identify nonpolar reference structures for existing polar materials^7–9^. Other studies have used a combination of group theoretic and first-principles calculations to propose ferroelectric candidates^10–12^. Bennett and co-workers proposed using high-throughput calculations to perform chemical substitution into structures of known classes of ferroelectrics^13–15^. Recent work used high-throughput phonon calculations to identify ferroelectrics through polar soft phonon modes of nonpolar phases^16^.

Previous studies have focused on a limited number of compounds or families of compounds using a relatively narrow set of symmetry conditions. Current curated lists of ferroelectrics only include known ferroelectrics that have been experimentally verified. With shrinking computing costs, high-throughput material searches using first-principles methods provide an efficient strategy to discover and catalog materials. Ferroelectric databases and systematic screening of properties such as band gaps, polarizations, volume expansion, critical temperatures, and coupling to magnetic and/or topological degrees of freedom may lead to new functional materials and potentially new physical phenomena.

In this work, we integrate density functional theory (DFT), crystal structure databases, symmetry tools, workflow software, and a custom analysis toolkit to build a workflow capable of generating libraries of known, previously-proposed and newly-proposed ferroelectrics. This workflow is general and can be used with any crystal structure dataset. We present the results from performing this workflow on the Materials Project database of inorganic crystal structures^24^. We screen over 67,000 material structures using symmetry relations between nonpolar and polar structure pairs and calculating the polarization from first-principles calculations. We identify 255 ferroelectric candidates, 200 being classified as high-quality candidates by a stringent verification process. Within these high-quality candidates, 74 are known or previously proposed, and 126 are new ferroelectrics. With the workflow developed here, we construct the first automatically-curated first-principles dataset of diverse, multi-class known and new ferroelectrics calculated with a standardized method that permits straightforward comparison. This dataset can be used to develop new tools and criteria for studying ferroelectricity across diverse materials systems. In addition our code for conducting this search has been contributed to the open-source python packages atomate and pymatgen so others can conduct searches of their own and build directly on this work^37,38^.

Our automated workflow has three stages: symmetry analysis, first-principles calculations, and post-processing. Accordingly, the rest of the work is organized as follows: the description of the workflow, based in the concept of ferroelectric nonpolar-polar symmetry pair, is described in the Methods section. Technical aspects of our workflow and database are included in the section Data Records. Finally, we validate our workflow method against experimental databases of ferroelectrics and verify our workflow is comparable to previous first-principles results in the section Technical Validation.

## Methods

### Identifying ferroelectricity from first principles

Ferroelectrics are characterized by a polarization versus electric-field hysteresis loop. Experimentally, the spontaneous polarization can be determined as half of the change in polarization at zero external field^31^. The spontaneous polarization is not a direct observable; one measures the change in spontaneous polarization between two stable configurations of a material^39^.

In three dimensions, the only space groups compatible with a polarization, meaning they leave a vector invariant under its symmetry operations, are those with polar point groups. Out of the 32 crystallographic point groups, 10 are polar; these polar point groups can keep points along specific lines (point groups *2*, *3*, *4*, *6*, *mm2*, *4mm*, *3m*, *6mm*), planes (point group *m*), or all points in three-dimensional space (point group *1*) invariant^40,41^. All other point groups are nonpolar. We define polar structures as crystal structures with a polar space group, which is composed of a polar point group plus translations, likewise for nonpolar structures.

Following the modern theory of polarization^39,42–47^, the polarization, **P**, of a crystal is defined as,

1 $${\bf{P}}={{\bf{P}}}_{0}+\sum _{i\in \{a,b,c\}}{n}_{i}\frac{e{{\bf{R}}}_{i}}{\Omega },$$

where *e* is the charge of the electron, ${n}_{i}$ is an integer, ***R***_*i*_ is a primitive lattice vector, and Ω is the unit cell volume (bold letters denote vectors). **P**_0_ includes electronic and ionic contributions. The second term on the right hand side of Eq. 1 is the quantum of polarization, $e{{\bf{R}}}_{i}/\Omega$, a consequence of translation symmetry. In the general case, **P**_0_ is defined up to any integer multiple of $e{{\bf{R}}}_{i}/\Omega$; for nonmagnetic crystals containing elements from even columns of the Periodic Table, **P**_0_ is defined up to even integer multiples. Since we are screening many systems here, we use the more general definition in this work.

Only differences of the computed polarization on the “same branch” are physically meaningful, where different branches are related by integer multiples of the quantum of polarization. Equivalently, the evolution of the polarization along an adiabatic path between two states must be smooth. Nonpolar space groups can only host formal polarizations that are zero or one-half modulo the polarization quantum. We use a nonpolar reference structure to calculate the change in polarization due to a polar distortion. In general, paths between two opposite polar configurations are sufficient to compute the change in polarization; however, we choose to use a nonpolar reference structure in this work as is standard for calculating the polarization from first principles.

To recover a smooth polarization path, we ensure the nonpolar structure must be continuously deformable into the polar structure along a path that preserves the symmetry of the polar structure and for which the system remains insulating. We then perform calculations of multiple structures along the distortion path to compute the spontaneous polarization, which can be compared to experiment. Using this approach, the spontaneous polarization can be directly predicted using first-principles methods with good accuracy^48^.

Hence, for the purposes of this work, we only consider ferroelectrics for which a high-symmetry nonpolar reference structure can be readily identified in the database for a lower-symmetry polar structure that can support a polarization^42^. We automate a search for compounds supporting two such phases and then compute the polarization difference along the structural path connecting the two structures.

If a polar ferroelectric structure corresponds to a metastable state, and is higher in energy than a nearby non-polar ground state by a small amount, the system can be considered an antiferroelectric^49,50^. Antiferroelectrics exhibit double hysteresis loops in polarization versus electric-field measurements; the field-induced first-order phase transition originates with an energy barrier between the nonpolar ground state and the polar metastable phase. In an antiferroelectric, the nonpolar ground state phase is related by a nonpolar distortion to a distinct nonpolar reference structure; the ground state structure is generally characterized as “anti-polar” to distinguish it from the nonpolar reference structure. Symmetry conditions for antiferroelectrics are described in ref. ^51^. For completeness, we note that to identify antiferroelectrics using the workflow presented here, in addition to finding a reference nonpolar phase, the polar metastable ferroelectric phase and an antipolar ground state, the material would need to display a small energy difference between the polar and antipolar structures on the order of 1–10 *meV*^52^.

### Workflow overview

**Fig. 1:**
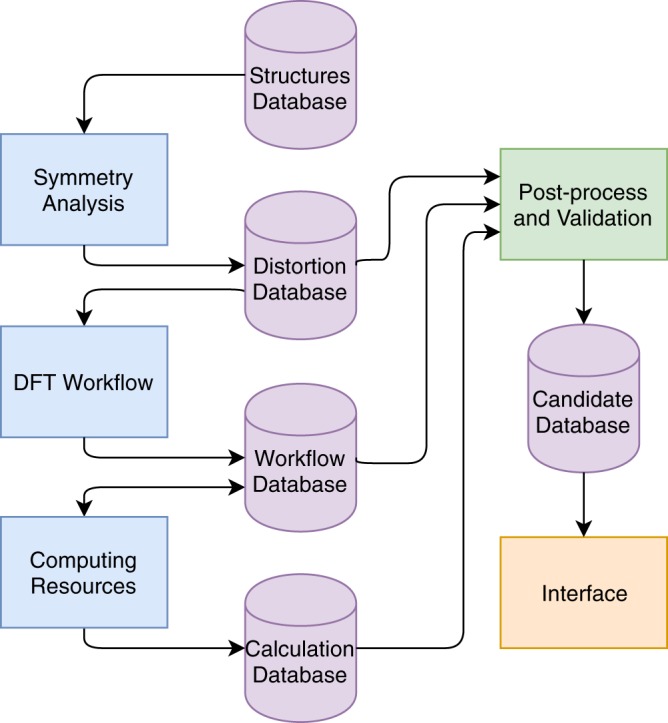
Diagram of the automated ferroelectric search workflow developed here. Databases are shown as purple cylinders. Processes are shown as rectangles: blue designates processes used to identify and perform first-principles calculations, green designates post-processing and verification, and orange designates the web-interface. Arrow directions indicate the flow of information. For example, the Workflow Database provides information to the Computing Resources about which calculations to compute and the Workflow Database is updated as calculations complete or as errors occur on the Computing Resources.

We first describe the general workflow diagram comprising symmetry analysis, first-principles calculations, and post-processing. As shown in Fig. 1, the complete workflow involves the passing of data between many separate calculations. In developing our workflow, we automate the following tasks:

1. Identifying candidate materials possessing nonpolar-polar structure pairs related by a continuous distortion (an isotropy subgroup symmetry relation).
2. Performing spin-polarized DFT calculations of changes in total energy, band gap, and polarization for multiple structures along the nonpolar-polar distortion.
3. Post-processing calculation data to compute the spontaneous polarization of the polar ferroelectric phase.
4. Validating the calculation quality for each ferroelectric candidate.
5. Creating an interface for viewing the results for all candidates (see the section Graphical Interface).

We start by choosing a crystal structure database on which to perform the search (see Structure Selection Symmetry Analysis). We emphasize that any crystallographic database (e.g. any of the databases described in^24–30^) can be used to perform our workflow, as long as the atomic coordinates and lattice parameters of the structures are provided.

Within the chosen database, we perform a symmetry analysis to find candidate materials possessing nonpolar-polar structure pairs related by a continuous symmetry deformation. Any such pairs found to satisfy the symmetry deformation criteria are stored in the Distortion Database as being deformable by symmetry. This criteria includes the following conditions: (1) The polar structure belongs to a space group that is a subgroup of the space group of the nonpolar structure; and (2) There exists a transformation matrix between the high-symmetry setting of the nonpolar structure to the low-symmetry setting of the polar structure. The latter imposes that the distortion of the lattice parameters and atomic coordinates between the nonpolar and polar structures is continuous, meaning the polar structure belongs to an isotropy subgroup of the nonpolar structure.

We then carry out DFT calculations on the candidate pairs to extract the changes in the band gaps, total energies, and polarization along the nonpolar-polar distortion (see Computational Methods). These results are stored in a Workflow Database and then accessed by our Computing Resources to perform the calculations. Next, the information stored in the Distortion, Workflow, and Calculation Databases is used together to post-process quantities such as the computed spontaneous polarization and to validate ferroelectric candidates using experimental and previous first-principles results (see Post-processing and Spontaneous Polarization Values and Verification of Computational Methodology). The information needed to assess the quality and properties of the candidates is then added to the Candidate Database where it can be accessed by our web Interface for viewing the candidate materials in aggregate (see Graphical Interface). Finally, candidates are screened to ensure the polarization and energy profile across the nonpolar-polar distortion are smooth and continuous, i.e. all calculations ended correctly and provide reliable results.

In the sections below, we describe in detail the methods used for creating an automatically curated dataset of ferroelectrics from the Materials Project database^24^. The Materials Project database is largely based on structures from the Inorganic Crystal Structure Database (ICSD)^27,28^ and includes hypothetical structures created through stoichiometric substitution. We use the Materials Project database to test our workflow. Our results for the Materials Project are not intended as the most general curated list of ferroelectrics; however, as the first automatically obtained list of ferroelectrics, they uncover new candidates and provide a blueprint for further studies. More elaborately curated lists may be constructed by applying our workflow to additional databases in future studies. The results from applying our workflow are described below and summarized in Table 1. We note that our workflow is modular and open-source, so it can be readily adapted and applied by others to expand the search for ferroelectrics and related materials such as antiferroelectrics and multiferroics.

**Table 1:** Results obtained by applying our workflow to the Materials Project database.

Symmetry	Structures^†^	∼67,000
	Polar structures^†^	∼15,000
	Distinct polar formula^†^	∼10,000
	Nonpolar-polar structure pairs	∼17,000
	Structure pairs with distinct chemical formulae	∼1,600
	Pairs with continuous transformation	413
DFT	Pairs with metallic endpoints	80
	Pairs with metallic interpolations	59
	Pairs with calculation errors	19
	Pairs that completed successfully	255
Valid	High-quality ferroelectric candidates	200
	Known ferroelectrics in high-quality candidates	74
	New ferroelectrics in high-quality candidates	126

The symbol ^†^ indicates “in Materials Project database at time of search”. Boxes relate numbers by symmetry conditions (Symmetry), first-principles calculations (DFT), and validation processes (Valid.). “Nonpolar-polar structure pairs” satisfy simple group-subgroup relations while “pairs with continuous transformation” satisfy group-isotropy subgroup relations.

### Structure selection

As motivated earlier, the input to our workflow is a collection of candidate materials possessing nonpolar-polar structure pairs. There are several methods that can be used to create candidate nonpolar-polar structure pairs. For example, one can apply a polar distortion to an existing nonpolar structure or create a hypothetical nonpolar reference structure for an existing polar structure. In this work, to classify a material as a ferroelectric candidate, we require both nonpolar and polar structures to be present in the database. As shown below, even this direct approach provides new candidate materials, previously overlooked as ferroelectrics. Future studies may choose to relax this requirement to identify a greater number of promising materials.

To search for compatible nonpolar-polar structure pairs in the Materials Project dataset, we first select compounds possessing nonpolar and polar structures with space groups which are related by a group-subgroup relation. Note that in principle the same compound may display more than one ferroelectric structural transition, and therefore have more than one nonpolar-polar symmetry pair. For each of these pairs, we require that the number of sites in the nonpolar structure is less than or equal to the number of sites in the polar structure. We perform this initial query using pymatgen, spglib, and the Materials Project API^37,53–55^. We provide the number of nonpolar-polar structures pairs resulting from this query in the top box of Table 1.

At the time of this search, the Materials Project database had approximately 67,000 structures, approximately 15,000 of which are polar. We find approximately 17,000 nonpolar-polar structure pairs related via group-subgroup space group relations. This number is large, in part, because the same polar structure may be paired with multiple possible nonpolar reference structures, and vice versa. This number is also large because the requirement that the polar structure is in a subgroup of the nonpolar space group is a much weaker requirement than the polar structure belongs to an isotropy subgroup of the nonpolar structure–which we check later in the workflow. These roughly 17,000 pairs contain approximately 1,600 of the approximately 10,000 distinct polar compositions in the Materials Project database. The remaining polar compositions in the Materials Project do not have symmetry compatible nonpolar structures within the same composition present in the database. We note that it is possible to propose hypothetical nonpolar reference structures for polar candidates using group theoretic methods or by relaxing the symmetry tolerance between nonpolar-polar distortions^7–9,16^; this is left for future work.

#### Naming conventions

We adopt the pymatgen alphabetical_formula method for the Composition class (with spaces and 1 s removed) to output consistent formulas for our candidates. We note that this method orders elements in such a way that does not match conventions in the literature. For example, we use O_3_ PbTi where the standard in the literature is PbTiO_3_. Compositions printed by pymatgen also differ from those used in mineralogy, such as for boracite, lawsonite and many other minerals in our dataset. In our datafiles, we also provide formula name output using the pymatgen reduced_formula method for the Composition class, which sorts elements by electronegativity.

### Symmetry analysis

The automated nature of our ferroelectric search relies on strict symmetry criteria. As described in the Structure Selection section, we pre-screen our candidate nonpolar-polar structure pairs using the symmetry tools in pymatgen and spglib to ensure that these pairs satisfy preliminary group-subgroup relationships. We then use the Structure Relations symmetry tool provided by the Bilbao Crystallographic Server (BCS)^56–58^ to impose the symmetry criteria described in the Workflow Overview, namely, to obtain a transformation matrix connecting the lattice parameters and atomic coordinates of the structure pair^59,60^. The BCS has a freely available web interface for accessing a wide variety of symmetry tools. We create python scripts to automate interaction with and scrape returned data from the BCS to perform our symmetry checks using the python package mechanize^61^. The Structure Relations tool checks the following:

1.1 The group-subgroup index relations are compatible. The index of a group-subgroup relation indicates the number of ferroelectric domains (distinct polar variants) that arise from the symmetry breaking of the high-symmetry structure.

1.2 There exists a path of maximal subgroups between the high-symmetry structure and low-symmetry structure.

1.3 The Wyckoff position splitting of the high-symmetry structure is compatible with the Wyckoff positions of the low-symmetry structure.

1.4 The lattice of the high-symmetry structure in the low-symmetry setting must be within a defined tolerance of the lattice of the low-symmetry structure (see Symmetry Precision section).

1.5 Each atom in the high-symmetry structure in the low-symmetry setting can be paired to an atom in the low-symmetry structure such that atom pairs are separated by a distance no greater than a given tolerance (see Symmetry Precision section).

Structure Relations takes Crystallographic Information Files (CIFs) of high-symmetry and low-symmetry structures and tolerance threshholds as arguments. We use a lattice tolerance of 3 Å and 10° for lattice parameters and angles, respectively. These tolerances are generous for materials with average sized unit cells (i.e.with lattice parameters less than 20 Å) and permit a wide variety of distortions. For the present work, high-quality candidates are reported for a maximum pairing distance of 1.5 Å. As shown in Table 1, out of the 17,000 structure pairs that we test with Structure Relations, 413 are found to be deformable by symmetry with a maximum distortion less than or equal to 1.5 Å.

#### Symmetry precision

Symmetry precision is a tolerance factor used to assess whether an atom is equivalent to another after a symmetry operation up to a maximum distance. A symmetry precision between 10^−1^ and 10^−5^ Å is typically used. In the Materials Project database, a symmetry tolerance of 10^−1^ Å is used for the reported space group stored in the database. We use the same tolerance to generate CIFs sent to the BCS Structure Relations.

We evaluated how varying the symmetry tolerance changes the resolved space group for all the structures in the Materials Project. We were able to determine this efficiently by using a binary search on a log_10_ scale for a maximum and minimum symmetry tolerance of 10^−1^ Å and 10^−5^ Å, respectively. Out of the 67,000 structures we checked, 50,000 (75%) structures were resolved into one distinct space group for the entire symmetry precision range. For additional discussion about the sensitivity of symmetry precision on resulting space groups in the search for ferroelectrics, see refs. ^16,62^.

### DFT calculation details

In our workflow, we perform spin-polarized DFT calculations using the Vienna Ab initio Software Package (VASP) version 5.3.5^63–65^. We use the generalized gradient approximation (GGA) functional of Perdew, Burke, and Ernzerhof (PBE)^66^. Our calculations use PAW pseudopotentials and an energy cutoff of 520 eV for the plane-wave basis^67,68^; this is 1.3 times the highest cutoff recommended for the pseudopotentials used^69^. Structures are initialized with ferromagnetic ordering in all cases in this work. Since the default is for parallel alignment of the spins, we expect the workflow to be reliable for nonmagnetic or ferromagnetic materials. Materials with more complicated magnetic ground states, such as antiferromagnets, would require consideration of different antiferromagnetic orderings. Therefore, some common multiferroics possessing antiferromagnetic or near antiferromagnetic ordering may not be identified by this workflow. Extensions to include antiferromagnetic spin arrangements are relegated to future work. These settings correspond to default values used to create the Materials Project database and therefore allow a direct comparison of our results with the Materials Project database.

We use the Berry phase approach from refs. ^39,43,44,70,71^, as implemented in VASP to calculate the electronic part of the macroscopic spontaneous polarization. We calculate the ionic part of the polarization using the point charge and position for each atom in the unit cell, see the calc_ionic function in pymatgen.analysis.ferroelectricity.polarization for details.

We use the default parameters for VASP inputs as defined in pymatgen (and used by Materials Project) and atomate^69,72,73^. For details on these parameters, see the documentation for pymatgen.io.vasp.sets.MPStaticSet. We use a Hubbard U correction to correct the DFT-PBE description of *d* states of select oxides and fluorides following the approach in ref. ^74^. To see the guidelines for which compounds we apply a U, see refs. ^24,69,72,73^. We use a reciprocal **k**-point density of 50 **k**-points per (1/Å)^3^ for structural relaxations and 100 **k**-points per (1/Å)^3^ for static and polarization calculations. We use total energy convergence criteria of 5 × 10^−5^ eV per atom for the electronic self-consistent loop and 5 × 10^−4^ eV/Å per atom for the ionic relaxation loop for structural optimizations. These convergence parameters were tested against higher-accuracy convergence parameters on a set of 182 chemically diverse compounds in ref. ^73^, yielding total energies within 15 *meV*/atom and lattice volumes within 7.5% of the higher-accuracy calculations for nearly 96% of the compounds.

We note that while the local density approximation (LDA) is commonly used to describe certain ferroelectric oxides, and therefore to compute their polarizations, use of a generalized gradient approximation (GGA), such as the PBE functional, tends to be standard for wider classes of materials nowadays. PBE is also the default functional used by the Materials Project for structural relaxations and calculating material properties. Thus, we use PBE for this effort. Our results are in line with the typical overestimation of PBE for the lattice parameters, and therefore, we expect a similar overestimation of the polarization. In addition, while DFT-PBE tends to underestimate electronic band gaps, the latter plays a minimal role in the determination of standard ferroelectric materials, and as shown below, will only limit the computation of the spontaneous polarization for a small number of them.

### Scientific workflow packages

We construct the scientific workflows to perform the structural relaxations and spin-polarized DFT calculations of energy, band gap, and polarization using the FireWorks and atomate python packages^38,75^. FireWorks is built for managing computational scientific workflows. atomate is built for constructing workflows for multiple computational material science codes, such as VASP. atomate uses FireWorks classes to develop modules for performing common DFT calculations with VASP, such as structural relaxations and self-consistent calculations of total energy. atomate also provides a framework for building custom modules, which we use to construct our structural interpolations and polarization calculations modules.

### DFT workflow

**Fig. 2:**
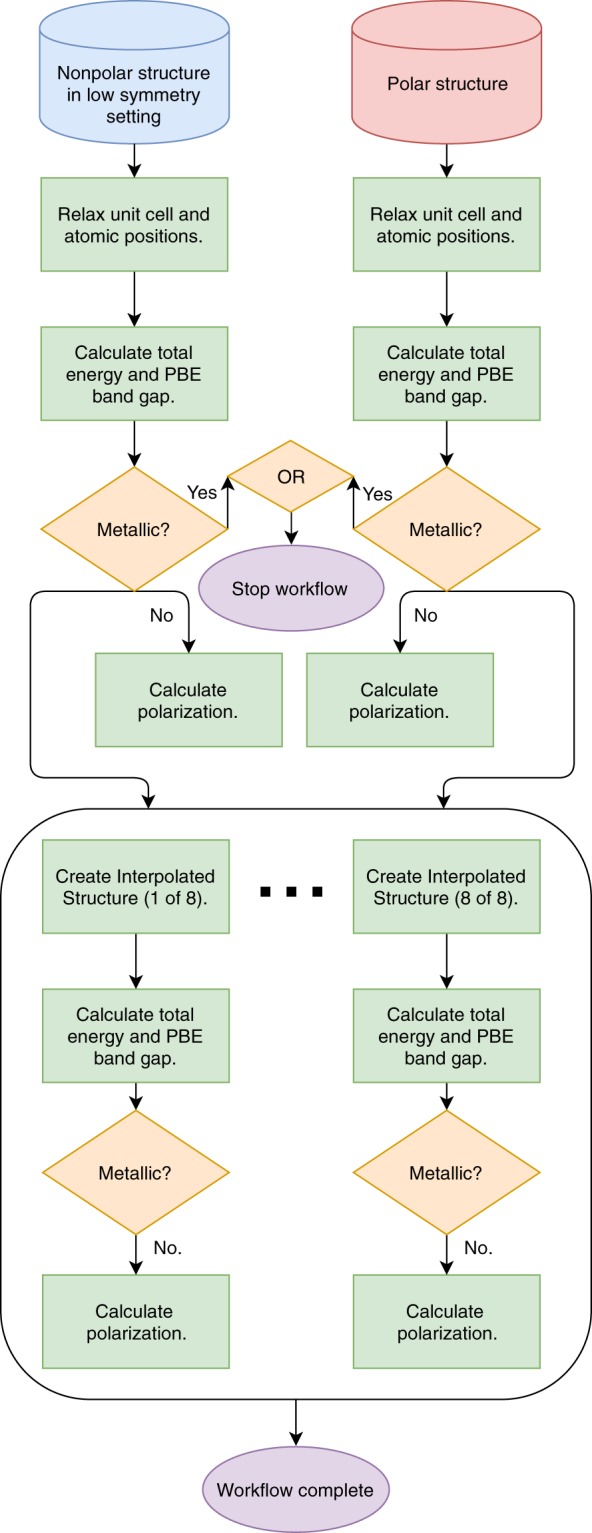
Diagram of DFT workflow written with atomate and Fire Works. Blue and red boxes denote initial nonpolar and polar structures, respectively, green boxes denote DFT calculations, orange rhombuses denote decision steps, and purple ellipses denote exit steps. Black arrows represent passing of data between different software codes. The metallic check for the interpolated structures leads to a similar condition as earlier, where if any of the interpolations are metallic the workflow stops (not illustrated for clarity in figure).

We use the DFT workflow, shown in Fig. 2, to compute the physical properties needed to identify ferroelectric candidates. We perform spin-polarized calculations and for systems with spin-polarized ground states, consider only ferromagnetic ordering. We execute the DFT workflow shown in Fig. 2 for the 413 pairs with continuous nonpolar-polar transformations, with maximum distortions that do not exceed 1.5 Å.

For each structure pair, we begin with the nonpolar structure in the low-symmetry setting (obtained from BCS Structure Relations in the Symmetry Analysis step) and the polar structure. We use the nonpolar structure transformed into the low-symmetry setting so we can perform structure interpolations and polarization calculations across similar lattices. We perform relaxations of the unit cell and atomic positions of both of these structures twice, using a level of convergence similar to what Materials Project uses for its database entries. As mentioned, all calculations are spin-polarized and use PBE, with a Hubbard U correction for systems with non-*d*^0^ open-shell cations; our workflow assumes ferromagnetic ordering for all systems. Extensions of our workflows to consider antiferromagnetic and other orderings will be the subject of future work. We then fix the relaxed nonpolar and polar structures, and perform a self-consistent DFT calculation to compute the total energy and band gap. If either the nonpolar structure or polar structure is found to be metallic at the DFT-PBE level in our spin-polarized calculations initialized with ferromagnetic orderings and a standard U–here we define metallic as having a DFT-PBE band gap of less than 10 *meV*–we stop the workflow for that structure pair.

If the polar and nonpolar structures are both insulating, we compute the polarization along the distortion path. As shown in Table 1, 80 of the 413 structure pairs were computed to have metallic endpoints in our spin-polarized calculations: 30 were found to have a metallic nonpolar structure but insulating polar structure, 2 were found to have a metallic polar structure but insulating nonpolar structure, and 24 were found to have both metallic polar and nonpolar structures. 24 additional structures have at least a metallic nonpolar structure, but these workflows were halted before the polar structures had their band gaps computed.

We compute the DFT total energy, band gap, and polarization of eight evenly-distributed linearly-interpolated structure, or interpolations, of the nonpolar to polar structures. We found eight interpolations to be sufficient for reconstructing a smooth polarization trend for at least 75% of our candidates. Similiar to the previous step where a metallic calculation causes the workflow to stop, metallic interpolations similarly halt the workflow since we would be unable to calculate the polarization of that structure. 59 candidates were found to have metallic interpolations. The DFT workflow is labeled as complete when all polarization calculations along the path have completed. As shown in Table 1, 255 structure pairs successfully completed the workflow, and satisfy our requirements of a ferroelectric phase transition.

### Post-processing spontaneous polarization values

As discussed earlier, only polar space groups are compatible with a polarization vector that is not integer or half integer multiples of the polarization quantum^46^. If a reference nonpolar structure that is continuously deformable into the polar structure can be identified, we can then calculate the polarization of several interpolated structures between the reference nonpolar and the target ferroelectric polar structure. The nonpolar structure is used as a means to calculate the spontaneous polarization; however, we note in general it is not necessary for the nonpolar structure to be experimentally observable for the polar material to be ferroelectric.

We start by calculating the formal polarization of the nonpolar structure, which is either zero or a half quantum of polarization (modulo the quantum of polarization) along the three lattice directions. Then we perform the same calculation for the first interpolated structure along the distortion, and then the next, until we arrive at the final polar structure. For a sufficient number of interpolations between the nonpolar and polar structures, we can trace out smooth, continuous polarization “paths” along the distortion; there will be infinitely many paths due to the periodicity of the polarization lattice. Subtracting the polarization values at the nonpolar and the polar endpoints of the same path or “branch” will give us the spontaneous polarization vector of the polar ferroelectric phase.

**Fig. 3:**
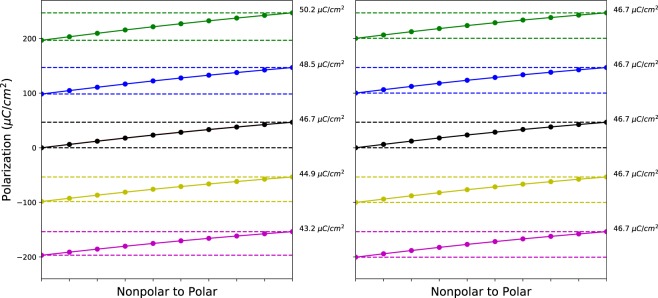
Examples of improper (left) and proper (right) polarization curves for BaTiO_3_ along the ${\hat{c}}$ direction versus distortion from the nonpolar to polar structure, showing the importance of calculating the proper polarization. Due to the change in lattice parameters and volume across the distortion, the quantum of polarization defined for each structure along the ${\hat{c}}$ direction is different. Using these different quanta causes the improper spontaneous polarization predicted by different branches to differ, as can be seen in the polarization values given in the right of the image. In contrast for the proper polarization (right), we re-scale the polarization of each intermediate structure to be in the polar structure’s polarization lattice and use the quantum of polarization as defined by the polar structure. This results in predictions that are branch independent, which is what we use to assess candidates. Note that while, in this specific case, the calculated polarization values for all interpolations were on the same branch, this need not generally be the case.

We perform the following steps to recover the same branch “proper” polarization, which is independent of choice of branch. The first step, which is crucial, is to readjust the polarization for each structure along the distortion to be in the polar polarization lattice. To do this, we modify the polarization of the intermediate structures by the ratio of the quantum of polarizations of the two lattices (the lattice parameters divided by the volume multiplied by the electron charge), i.e.,

2 $${P}_{\eta ,i}^{{\rm{(polar}}\,{\rm{lattice)}}}={P}_{\eta ,i}\left(\frac{{R}_{{\rm{polar}},i}{/\Omega }_{{\rm{polar}}}}{{R}_{\eta ,i}{/\Omega }_{\eta }}\right),$$

where ${R}_{\eta ,i}$ and ${\Omega }_{\eta }$ are the lattice parameters and volume of the ${\eta }^{th}$ structure along the distortion, ${R}_{{\rm{polar}}}$ and ${\Omega }_{{\rm{polar}}}$ are the lattice parameters and volume of the polar structure, and *i* is a lattice direction ${ {\hat{a}} }$, $\widehat{b}$ or ${\hat{c}}$. If we do not perform this adjustment, we are calculating what is called the “improper polarization” which will depend on the choice of branch and is therefore unphysical^76^. See Fig. 3 for an example of the differences between proper and improper polarization for BaTiO_3_ (here we use conventional notation for the name).

After adjusting our initial polarizations, we then construct a periodic lattice with lengths and angles corresponding to the quantum of polarization along each polar lattice direction; this corresponds to the second term of Eq. (1). For the polarization lattice, the lengths of the lattice vectors are the cell lattice vectors divided by the volume of the unit cell and multiplied by conversion factors for electron charge and length scale.

**Fig. 4:**
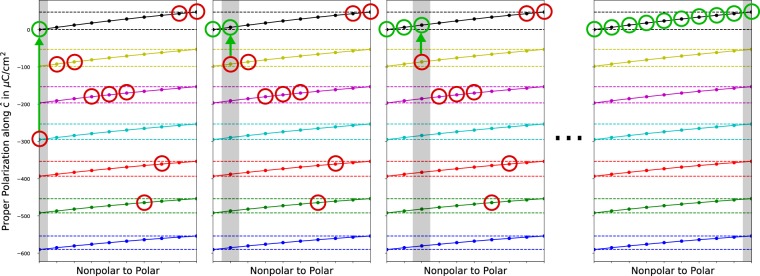
A visual demonstration of the same branch polarization algorithm demonstrated in one-dimension (rather than three-dimensions) using BaTiO_3_. The values for the polarization for each interpolation are those circled in red. In the first panel, we adjust the nonpolar polarization to be on the branch closest to zero. In the second panel, we move the first interpolated polarization to be on the branch closest to the adjusted nonpolar polarization. In the third panel, we adjust the second interpolated polarization to be on the branch closest to the first interpolation polarization. If the algorithm finishes successfully, all the adjusted polarizations will be on the same branch.

Our algorithm for adjusting the polarizations to be on the same branch is depicted in Fig. 4. First, we take the nonpolar polarization (adjusted to be in the polar polarization lattice), and choose the “image” (or periodic value) of the polarization value in the nonpolar polarization lattice that is closest to the Cartesian origin (0, 0, 0). The value of the nonpolar polarization along ${ {\hat{a}} }$, $\widehat{b}$, and ${\hat{c}}$ can either be zero or a half-quantum. Then, we find the image of the first interpolation polarization value (again adjusted to be in the polar polarization lattice) that is closest to the Cartesian coordinates of the adjusted nonpolar polarization value. We continue this process until we get to the polarization of the polar structure.

**Fig. 5:**
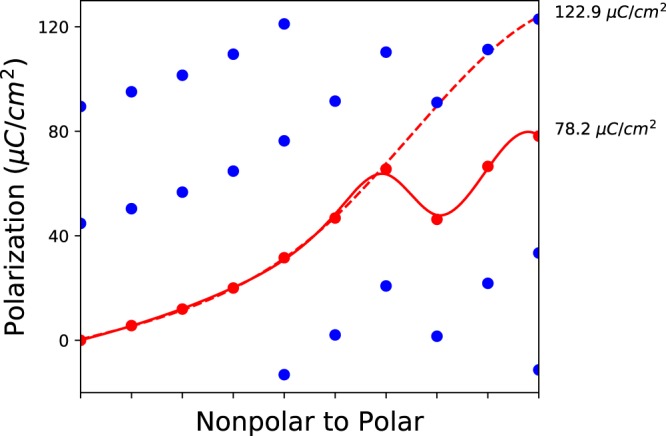
The polarization reconstruction for CrO_3_ with search ID 187. The polarization along the *b* lattice parameter is incorrectly reconstructed because the different polarization branches are closer than the change in polarization between structure interpolations.

This algorithm will find the polarization path with the smallest difference between polarizations of subsequent interpolations. An issue is that this algorithm can incorrectly find the same branch polarization in cases where the change in polarization between interpolations is larger than the quantum of polarization between branches. One example of this type of failure is the polarization resolved for CrO_3_ with search ID 187, see Fig. 5 (the search ID being a simplified unique identifier, defined for the purposes of our work, for pairs of structures used in our workflow search, see Online-only Table 3). In this example, the algorithm chooses a discontinuous path that has a smaller spontaneous polarization of 78.2 *μC*/*cm*^2^ in red. However, the correct path uses the last three interpolations in the branch shown with a dashed red line and gives a polarization of 122.9 *μC*/*cm*^2^. To correctly reconstruct this polarization with our existing algorithm, more structures would be needed to better interpolate between the nonpolar and polar structures. Alternatively, the curvature of the spline connecting nonpolar and polar structures could be used to identify points on the same branch; we leave this refinement for future work.

**Table 2:** Key, value data type, and value description for workflow_data.json entries.

Key	Type	Description
_id	bson.objectid.ObjectId	Automatically created unique identifier.
wfid	unicode	The workflow id.
cid	unicode	The “connection” or distortion id. The alphanumeric portion of the string after cid_ corresponds to the bson.objectid.ObjectId used in the distortion database.
search_id	unicode	Simplified unique identifier for pairs of structures used in the search.
workflow_status	unicode	Status of workflow denoted by FireWorks.
alphabetical_formula	unicode	Composition with elements sorted alphabetically.
pretty_formula	unicode	Composition with elements sorted by electronegativity.
polar_id	unicode	Materials Project Id.
nonpolar_id	unicode	Materials Project Id.
polar_icsd_ids	floats list	ICSD id numbers, if available.
nonpolar_icsd_ids	floats list	ICSD id numbers, if available.
polar_spacegroup	float	Polar space group, integer between 1 and 230.
nonpolar_spacegroup	float	Nonpolar space group, integer between 1 and 230.
orig_polar_structure	pymatgen.Structure dict	Polar structure as referenced in distortion JSON file.
orig_nonpolar_structure	pymatgen.Structure dict	Nonpolar structure as referenced in distortion JSON file.
structures	pymatgen.Structure dicts list	Static calculation structures. Fully complete workflows have 10.
relaxation_len	float	Number of relaxation calculations performed. Fully complete workflows have 2.
relaxation_task_labels	strs list	The task labels of the relaxation calculations performed.
static_len	float	Number of static calculations performed. Fully complete workflows have 10.
static_task_labels	strs list	The task labels of the static calculations performed.
polarization_len	float	Number of polarization calculations performed. Fully complete workflows have 10.
polarization_task_labels	strs list	The task labels of the polarization calculations performed.
polarization_change_norm	float	The Cartesian norm of the recovered spontaneous polarization.
polarization_change	floats list	The vector along a, b, and c of the recovered spontaneous polarization vector.
raw_electron_polarization	floats lists list	Raw electron polarization per structure from VASP along Cartesian directions.
raw_ionic_polarization_vasp	floats lists list	Raw ionic polarization per structure from VASP along Cartesian directions.
raw_ionic_polarization	floats lists list	Raw ionic polarization per structure from calc_ionic along lattice directions.
polarization_quanta	floats lists list	Structure dependent polarization quanta along a, b, and c lattice vectors.
same_branch_polarization	floats lists list	Same branch polarization along a, b, and c for each polarization calculation structure.
polarization_max_spline_jumps	floats lists list	Max jump between spline and data for polarization along a, b, and c.
polarization_smoothness	floats list	Average jump between spline and data for polarization along a, b, and c.

### Graphical interface

To view the DFT ferroelectric candidate data in aggregate, we create an interactive web site for viewing polarization and total energy plots, animations of the distortion, and other data. The interface consists of two main pages: (1) a page containing a sortable table of ferroelectric candidates organized by category (whether the candidate had a value of polarization successfully calculated and if so with what level of confidence) and (2) individual candidate pages that show energy and polarization plots, distortion animations, and other data specific to that candidate. This interface is available at https://blondegeek.github.io/ferroelectric_search_site/.

## Data Records

This dataset is available as two JSON files deposited in figshare^77^ and our GitHub repository (http://github.com/blondegeek/ferroelectric_search_site)^78^. The JSON files provide details of the symmetry analysis performed for each candidate and data generated by DFT calculations and post-processing from the workflow. Zipped folders of the input and output VASP files for each candidate deposited in figshare^77^. The title of the zipped folder includes the workflow ID to correlate the VASP files to information in the JSON files provided. We also provide an interface for viewing the dataset at http://blondegeek.github.io/ferroelectric_search_site with the code for the interface located at http://github.com/blondegeek/ferroelectric_search_site.

### File format

We contribute the following data:

1. JSON file with information on workflow status of each calculated candidate and calculation details extracted from VASP inputs and outputs. This includes total energy, band gap, polarization, post-processed information, and validation criteria for candidates with completed calculation. See Tables 2 and 3 for details.

**Table 3:** Key, value data type, and value description for workflow_data.json entries continued.

Key	Type	Description
energies	floats list	Energy in eV for each static calculation structure.
energies_per_atom	floats list	Energy per atom in eV for each static calculation structure.
energies_per_atom_max_spline_jumps	float	Max jump between spline and data for energy per atom.
energies_per_atom_smoothness	float	Average jump between spline and data for energy per atom.
calculated_max_distance	float	Calculated max distortion distance. Compare to dmax in distortion.json entries.
zval_dict	dict	dict with keys of species and values of ZVAL in number of electrons.
hubbards	dict	dict with keys of species and values of Hubbard U correction in eV pairs.
cbms	floats list	Conduction band minimum per static calculation computed structures.
vbms	floats list	Valence band maximum per static calculation computed structures.
stresses	floats lists list	Stress tensor per static calculation computed structures.
charges	floats dicts lists list	Charges projected onto spd orbitals per atom per static calculation computed structures.
magnetization	floats lists list	Magnetization in Bohr magnetons per atom per static calculation computed structures.
total_magnetization	floats list	Total magnetization in Bohr magnetons per static calculation computed structures.
forces	floats lists list	Cartesian forces per atom per static calculation computed structures.
bandgaps	floats list	list of band gaps in eV for static calculation computed structures.
2. JSON file with information describing all 413 nonpolar-polar structure pairs with group-subgroup relations compatible with a second-order phase transition in the Materials Project determined with BCS Structure Relations and used in this search. See Tables 4 and 5 for details.

**Table 4:** Key, value data type, and value description for distortion.json entries.

Key	Type	Description
_id	bson.objectid.ObjectId	These ids are used to generate cid in workflow_data JSON file.
pretty_formula	unicode	Composition with elements sorted by electronegativity.
polar_id	unicode	Materials Project Id.
nonpolar_id	unicode	Materials Project Id.
polar_icsd	float	ICSD id number, if available.
nonpolar_icsd	float	ICSD id number, if available.
polar_spacegroup	float	Polar space group, integer between 1 and 230.
nonpolar_spacegroup	float	Nonpolar space group, integer between 1 and 230.
bilbao_polar_spacegroup	float	Polar space group from Bilbao Crystallographic Server, integer between 1 and 230.
bilbao_nonpolar_spacegroup	float	Nonpolar space group from Bilbao Crystallographic Server, integer between 1 and 230.
distortion	dict	Details pertaining to distortion between nonpolar and polar structure.
polar_band_gap	float	Materials Project computed band gap.
nonpolar_band_gap	float	Materials Project computed band gap.

**Table 5:** JSON keys, value data type, and value description for distortion dictionary of distortion.json entries.

Key	Type	Description
high_symm	dict of pymatgen.Structure	Nonpolar structure in high-symmetry setting.
high_low_setting	dict of pymatgen.Structure	Nonpolar structure in low-symmetry setting.
low_symm	dict of pymatgen.Structure	Polar structure in low-symmetry setting.
high_pre	unicode	Structure information as directly output by Bilbao Crystallographic Server website.
high_low_pre	unicode	Structure information as directly output by Bilbao Crystallographic Server website.
low_pre	unicode	Structure information as directly output by Bilbao Crystallographic Server website.
distortion	list	Table of low-symmetry setting of Wyckoff position, the string “(x, y, z)”, species, distortion in x, y, and z, and the magnitude of distortion.
pairings	list	Wyckoff splitting pairing between high symmetry and low symmetry structures.
dmax	unicode	Maximum distortion distance between nonpolar and polar structure.
s	unicode	Degree of lattice distortion (S) is the spontaneous strain (sum of the squared eigenvalues of the strain tensor divided by 3).
dav	unicode	Maximum distortion distance between nonpolar and polar structure.
delta	unicode	The measure of compatibility (Δ) (Bergerhoff *et al.* 1998).
3. Zipped folders with the VASP 5.3.5 INCAR, KPOINTS, OUTCAR, and POSCAR files.

### Reported properties

For each candidate we provide an initial nonpolar-polar pair of structures, including the nonpolar structure in both the nonpolar, high-symmetry setting and polar, low-symmetry setting. We also provide the displacements of each atom and other metrics provided by BCS Structure Relations.

For each successful calculation, we provide the structure used for calculation, the ionic and electronic polarization computed by VASP, the ionic polarization computed via the method of point charges, the total energy and energy per atom of the structure, and other commonly computed quantities such as total magnetization, magnetization per atom, forces, and stresses. We also give details as to which calculations (out of the 22 computed) for a given candidate pair were completed.

For each set of completed calculations we also provide, the recovered spontaneous polarization using the workflow described in the Methods section, as well as spline data characterizing the smoothness of the polarization and energy trends across the nonpolar-polar distortion.

### Graphical representation of results

**Fig. 6:**
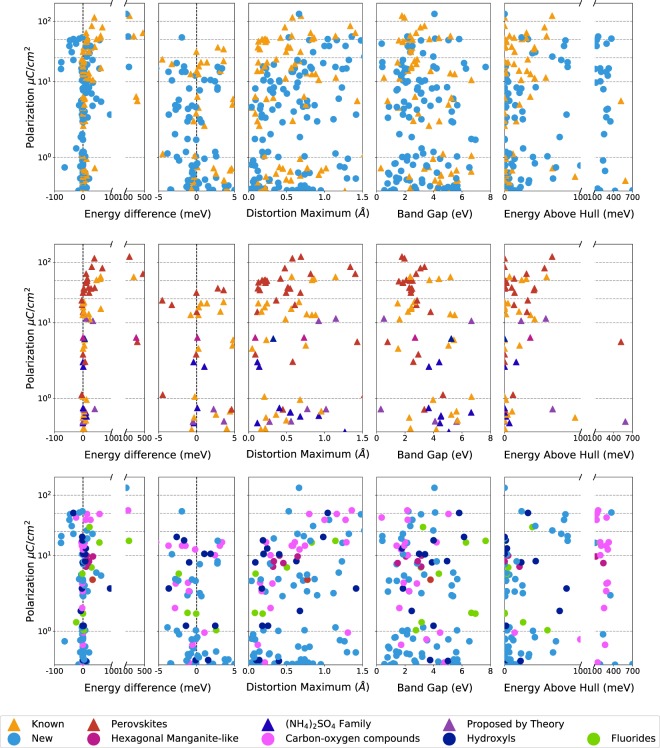
Validated ferroelectric candidates from our automated search in the Materials Project. Computed spontaneous polarization plotted against nonpolar-polar energy difference, maximum atomic distortion, PBE + U band gap, and energy above hull of the polar structure. All results are generated for spin-polarized DFT-PBE + U calculations. Note, that for spin-polarized systems, we only initialized the calculations with a ferromagnetic ordering. The energy above hull is extracted from the Materials Project. The legend labels different subcategories considered in this work and described in the text.

In the top row of Fig. 6, we partition the high-quality candidates found in the Materials Project into known and newly-proposed ferroelectrics and further partition those ferroelectrics into subclasses. In Fig. 6 we see that known and new ferroelectric candidates are well mixed along the metrics of nonpolar-polar structure energy difference, distortion maximum between nonpolar and polar structures, PBE band gap of polar structure, and energy above hull (a measure of the thermodynamic driving force for decomposition^79^).

In the middle row of Fig. 6, the candidates with large polarizations denoted with red triangles belong to the perovskite family. We recover many known perovskite ferroelectrics, such as: LiNbO_3_, AlBiO_3_, BiInO_3_, KNbO_3_, BaO_3_Ti, CdO_3_Ti and O_3_PbTi. In addition, we recover well-known double perovskite ferroelectrics, such as Bi_2_Nb_2_O_9_Pb and Bi_2_O_9_SrTa_2_. We also recover well-known antiferroelectrics possessing ferroelectric metastable phases, such as NaNbO_3_, HfO_3_Pb and O_3_PbZr. We again note that we do not use conventional notation for these systems but rather use alphabetical order of elements to provide a consistent ordering with our workflow output.

Other classes in the middle row of Fig. 6 are the organic (NH_4_)_2_SO_4_ family in blue and structures already proposed by theory to be ferroelectric in purple. These data show that there are many known and proposed ferroelectrics in the literature with polarizations of 10 *μC*/*cm*^2^ or less.

In the bottom row of Fig. 6, we categorize new ferroelectric candidates into different trending compositions or structure types. There are several candidates containing fluorides, carbon-oxygen compounds, and hydroxyl groups. We highlight these candidates because they are very different in composition from oxide ferroelectrics most common in the literature. We also point out some hypothetical non-magnetic hexagonal manganite-like structures found in the Materials Project database that have polarizations of approximately 10 *μC*/*cm*^2^ and half-quantum nonpolar polarizations.

**Fig. 7:**
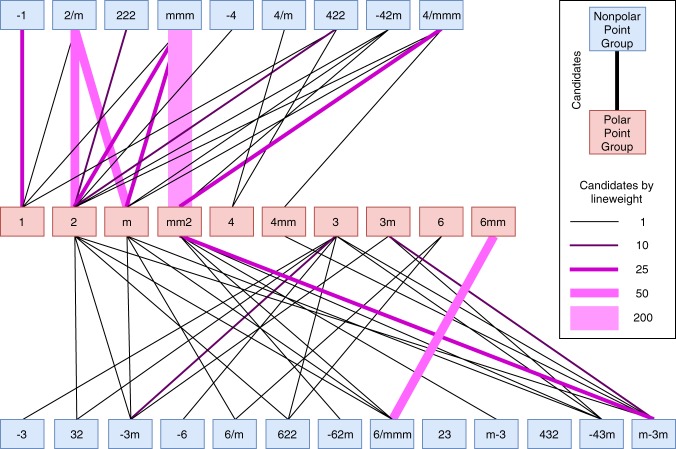
Schematic of the number of point group transitions between the 32 crystallographic point groups. The thickness and color of the line connecting nonpolar and polar point groups indicates the number of structures in the dataset with a continuous deformation between those point groups. The legend includes a schematic for nonpolar-polar phase transitions and describes the significance of line weights and colors connecting nonpolar and polar point groups.

In Fig. 7, we show trends in the number of pairs with continuous deformation with given nonpolar-polar point group transitions. There are 32 crystallographic point groups; nonpolar and polar point groups are shown in blue and red, respectively. The thickness and color of the line connecting nonpolar and polar point groups indicate the number of structures in the dataset with a continuous deformation between those point groups. We find that point group transitions that correspond to orthorhombic structures such as *mmm* → *mm2*, monoclinic structures such as *2/m* → *2* or *2*/*m* → *m*, and hexagonal *6/mmm* → *6mm* are the most prevalent.

**Fig. 8:**
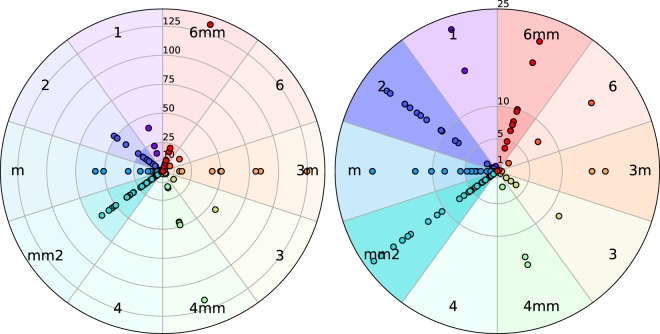
Diagrams of polarization magnitude vs. point group. The left diagram shows the full range of polarizations from 0 to 140 *μC*/*cm*^2^ and the right diagram zooms in on polarizations in the range 0 to 25 *μC*/*cm*^2^. Point groups are grouped according to symmetry. The darkness of a radial cell is proportional to the number of candidates in that region of the plot.

In Fig. 8, we show the computed polarization of the ferroelectric candidates plotted with respect to their polar point group, similar to the plot of piezoelectric tensor magnitudes in ref. ^4^. The majority of candidates have polarization less than 5 *μC*/*cm*^2^, shown on the right. The candidates in point group *4mm* with large polarizations are perovskites with a reference structure in point group $m\bar{3}m$. The degree of shading of a radial cell is proportional to the number of candidates in that region of the plot. For example, there are many candidates with polar point groups 2 and *mm2* that have polarizations within 25 *μC*/*cm*^2^.

## Technical Validation

### Verification of computational methodology

Several checks are needed to ensure our automated calculations have completed satisfactorily and the information automatically extracted from them is reliable. We describe these tests below.

#### Testing smoothness of energy and polarization trends with distortion

We flag any ferroelectric candidates whose calculations cannot be used to reliably assess the quality of the candidate. For example, if the trend in total energy is not continuous, we cannot be confident that we can extract a meaningful polarization trend. Similarly, if the same branch polarization is not continuous, we cannot be confident that an accurate spontaneous polarization has been determined.

To assess the smoothness of trends in polarization and energy across a distortion, we use UnivariateSplines from scipy.interpolate^80^. We use cubic splines for fitting polarizations and quartic splines for fitting total energies. We use the default smoothness parameter of 1.0. These splines are generated using the Polarization class in pymatgen.analysis.ferroelectricity.polarization.

We find that 31 out of the 55 materials that do not have smooth interpolations contain atoms with nonzero magnetic moments, mostly containing 3*d* elements (V, Cr, Mn, Fe, Co, Ni) and one containing the *5d* element W. We found 26 materials to have several discontinuities in total energy (even when these calculations resulted in smooth polarizations). These materials were transition metal oxides, fluorides, carbonates, orthosilicates, and phosphates with alkali or alkaline earth metals (Li, Na, and Ba for these specific examples), many being Li-ion battery cathode candidates. The transition metals in these materials (V, Cr, Mn, Fe, Co, Ni) can take multiple oxidation states. Because these discontinuities in energy were coincident with discontinuities in the total magnetization, we believe these jumps were caused by the transition metal species changing oxidation state through the distortion, suggesting the need to asses their ground state magnetic ordering more carefully.

#### Metallic endpoints and metallic interpolations

Polarization calculations require a nonzero band gap along the distortion path. Therefore, workflows that have either polar or nonpolar structures that are calculated to be metallic are halted. In the workflow_data.json, these workflows are designated by a workflow_status of “DEFUSED”. Of the 413 pairs considered, 80 possess metallic endpoints and therefore interpolations were not performed. Occasionally, interpolated structures between two nonmetallic structure endpoints are metallic and there were 59 that had metallic interpolation structures. If any structure along the path from nonpolar to polar structure is metallic, the quality of automated analysis cannot be guaranteed. We include these candidates in our dataset, but they are noted as having a polarization_len (see Tables 2 and 3) of less than 10 or do not have a polarization_change_norm (in cases where some of the interpolated polarizations are None). If a candidate has a polarization_len equal to 10 and have a polarization_change_norm, all interpolated polarization calculations completed successfully.

We also note that the method used to determine whether a material is insulating differs for pymatgen and VASP. In our workflow, pymatgen determines the band gap by comparing the energies of the band edges. When VASP determines whether to proceed with calculating the polarization, it checks whether the material is insulating by checking the occupations of the band edges. Since the occupations can be sensitive to the choice of smearing and k-grid density, there are instances where using our default settings in the workflow leads to partial occupancies, while the band edge energies suggest the material has a finite gap; therefore VASP will not proceed with calculating the polarization whereas our workflow deems the material to be insulating. This tends to occur for materials containing 3*d* elements which, as discussed earlier in the context of magnetism, would require more careful calculations to reliably capture their properties, and therefore not necessarily expected to successfully complete the workflow.

#### Comparison of materials project to relaxed structures

Two structural relaxation calculations are already performed on all structures in the Materials Project. We perform additional relaxations of the unit cell and atomic positions to ensure total energy convergence. We found only less than 5% (10%) of our relaxed structures have lattice parameter differences of more than 3% (1%) from the original Materials Project structures. Because we perform relaxations of nonpolar structures transformed to the low-symmetry polar setting, we compare the relaxed nonpolar structure to the low-symmetry transformed structure output by BCS of the nonpolar structure from Materials Project.

#### Identifying high-quality candidates

In any high-throughput search, there are calculations that complete without errors and some that require further scrutiny to interpret its results. We deem as high-quality candidates those calculations where the polarization and total energy trends are smooth and continuous; we define this criteria in the following way: the maximum difference between the data and spline fitted to the same branch polarization must be less than 10^−1^ *μC*/*cm*^2^, and the maximum difference between the data and the spline fitted to the energy trend must be less than 10^−2^ *eV*.

As shown in Table 1, out of the 255 candidates, 200 pass through our stringent verification criteria and ensures the polarization and energy trends across the ferroelectric distortion are smooth and continuous. The remaining candidates are still valid candidates; we recommend checking the polarization and energy trends by hand as the algorithms used for analysis may not have reliably recovered the spontaneous polarization in these cases, as in Fig. 5. These high-quality candidates are further described in the section Determining Known and New Ferroelectrics. There are candidates included in this list with polarization values of zero, as computed within the accuracy of our calculations; we include these since they pass the above criteria and could be tuned to host nonzero polarizations such as by chemical substitution. As well, the polarization values may be very small but this is also true of some of the known ferroelectric candidates computed with our workflow, and so for the sake of completeness we also list these candidates. We also call the reader’s attention to the materials with magnetic elements. Since all materials are initialized with ferromagnetic ordering, we do not expect to reliably capture materials with other magnetic orderings (e.g. antiferromagnets); as such, materials with nonzero magnetic moments may not necessarily have been calculated using their true magnetic ground state and those results should be interpreted with caution.

### Comparison to DFT calculated polarizations from literature

We verify that our workflow reproduces the first-principles calculated polarizations for a variety of ferroelectrics in the literature. DFT calculated values for the ferroelectric polarization depends heavily on the structures and functional used in the calculation, and for magnetic systems, it will also be sensitive to magnetic ordering. For example, while in our work the endpoint structures are fully relaxed, other works constrain the relaxed polar unit cell to have the same volume as the experimental structure^81^; these calculated polarizations will be systematically smaller than ours due to PBE optimized structures having larger lattice parameters than experimental values.

We compare to literature where a fully optimized (unit cell, volume, and atomic positions) relaxation procedure is used. For clarity of comparison, we use the chemical formula used by these references rather than the alphabetical_formula that is convention for the rest of this work.

The ferroelectric first-principles literature is largely dominated by studies of perovskites. We compare to calculations for the perovskites BaTiO_3_, PbTiO_3_, LiNbO_3_, BiAlO_3_, CdTiO_3_, BiFeO_3_, and we include in our comparison the double perovskite SrBi_2_Ta_2_O_9_. These comparisons are summarized in Table 6.

**Table 6:** Comparison of this work to other first-principles studies of ferroelectrics, primarily perovskites.

Formula and Space Group		*a* (Å)	*b* (Å)	*c* (Å)	*P*_*s*_ (*μC*/*cm*^2^)
BaTiO_3_ (99)	ref. ^87^	4.005	—	4.210	43.5
	ref. ^88^	4.000	—	4.216	47.0
	This work (1)	4.000	—	4.224	46.7
	This work (2)	4.001	—	4.215	45.9
PbTiO_3_ (99)	ref. ^88^	3.844	—	4.767	125.5
	This work (3)	3.871	—	4.594	116.8
LiNbO_3_ (161)	ref. ^88^	5.203	—	14.110	84.7
	This work (4)	5.216	—	14.116	84.5
SrBi_2_Ta_2_O_9_ (36)	ref. ^89^	5.550	5.550	25.100	34.1
	This work (5)	5.602	5.614	25.520	36.9
CdTiO_3_ (26)	ref. ^90^^†^	5.250	5.387	7.570	21.0
	This work (6)	5.402	5.525	7.694	37.2
CdTiO_3_ (33)	ref. ^90^^†^	5.239	5.378	7.619	29.0
	This work (7)	5.360	5.494	7.812	34.8
BiAlO_3_ (161)	ref. ^91^^†^	3.840 (cubic)	—	—	75.6
	This work (8)	3.844 (cubic)	—	—	80.3
KH_2_PO_4_ (43)	ref. ^88^	10.800	10.710	7.110	5.5
	This work (9)	10.730	10.652	7.105	5.2

For this table, we use the chemical formula conventions used in the works we compare to. We compare to calculations using PBE + U unless otherwise specified. The polar space group is given in parentheses under the chemical formula. The symbol ^†^Indicates the reference being compared to used the Local Density Approximation (LDA) functional in their calculations. LDA polarization values tend to be smaller than polarization values calculated with PBE (which we use in this work) due to smaller predicted lattice parameters by LDA than PBE. The search ids for entries in the table are: (1) 69 (2) 70 (3) 389 (4) 331 (5) 80 (6) 146 (7) 147 (8) 23 (9) 257.

We note again here that because our workflow assumes ferromagnetic ordering, it will fail for many multiferroics since they tend to have other magnetic orderings, such as antiferromagnetic or noncollinear orderings. For example, two important multiferroics are BiFeO_3_ and YMnO_3_; the former is only captured to some extent by our workflow and the latter is not reported at all by our database. The standard polar phase of BiFeO_3_ features G-type antiferromagnetic ordering, and that for YMnO_3_ features A-type antiferromagnetic ordering. As the present version of our workflow initializes all calculations with ferromagnetic ground states for all magnetic systems, we do not expect to capture most multiferroics using the workflow as implemented for this search with the Materials Project database. Additionally, we note that the standard nonpolar reference structure for BiFeO_3_ is in space group 167 (R$\bar{3}$c); however, the R$\bar{3}$c structure of BiFeO_3_ is not in the Materials Project database. That the R3m and Pm$\bar{3}$m structures of BiFeO_3_ calculated in this workflow, with ferromagnetic ordering and the default U value, are found to be insulating is fortuitous and allows it to be reported as a high quality candidate. However, the BiFeO_3_ experimentally verified ground state structure, 161 (R3c), did not complete our workflow due to metallic interpolations. Conversely, YMnO_3_ is DEFUSED and does not proceed to the interpolations because its nonpolar structure is found to be metallic in our calculations. Extending the workflows to account for antiferromagnetic and other spin-ordered ground states, as well as to relax the constraint that a nonpolar reference structure exist in the database, will enable casting a wider net for known and new multiferroics, and will be the subject of future work.

### Determining known and new ferroelectrics

We distinguish known from new ferroelectrics in our workflow depending on whether or not a material has been reported in the literature as ferroelectric. Thus, we perform a literature review by hand for every considered candidate, and leave automating such literature searches to future work. We find that out of 200 high quality candidates, 74 are known or previously proposed ferroelectrics and 126 are, to our knowledge, new ferroelectric candidates.

In Fig. 6, we plot calculated spontaneous polarization versus nonpolar-polar total energy difference, maximum distortion distance, PBE band gap, and energy difference between the polar structure and convex hull reported in the Materials Project for known, proposed and new ferroelectric candidates. We also provide tables of the known and new candidates grouped by chemical formula and polar space group in Online-only Tables 1 and 2, respectively. Details connecting these candidate to specific workflow calculations are in Online-only Table 3.

As seen in Fig. 6, known and newly-proposed ferroelectrics display similar dispersion and overlap in the range considered. The middle row of Fig. 6 demonstrates the variety of known ferroelectric candidates that we are able to recover, from perovskites to candidates in the organic (NH_4_)_2_ SO_4_ family to candidates proposed by theory. The bottom row of Fig. 6 shows categories of new ferroelectric candidates we find in the Materials Project, some from previously known ferroelectric classes such as hexagonal manganite-like structures and less-studied categories such as fluorides, carbon-oxygen compounds, and crystals containing hydroxyl groups.

### Comparison to hand-curated list of ferroelectrics in the Pauling Files database

The Pauling File is a materials database accessible through SpringerMaterials^18^. In this database, there are materials tagged as ferroelectric and antiferroelectric. We use these tagged entries to validate whether the workflow is able to successfully identify diverse ferroelectrics by examining which tagged (anti)ferroelectrics complete the workflow. In the Pauling Files, there are 955 distinct compositions tagged as (anti)ferroelectric, 306 of which are pure (not doped) compositions. Out of 306 pure compositions, 95 of those compositions are included in the Materials Project as polar materials. This does not necessarily mean that the Materials Project database contains the same ferroelectric polar structure as referenced in the Pauling Files; rather, it simply means that there exists a polar structure in the Materials Project with the same composition as a tagged ferroelectric or antiferroelectric in the Pauling Files. 57 of these compositions have nonpolar-polar structure pairs in the Materials Project, 40 of which are found to have a continuously distortion by BCS Structure Relations. 32 of the 40 are successfully identified by the workflow as high-quality candidates, meaning the energy and polarization trends are smooth. Out of the 8 candidates that did not successfully make it through the workflow as high quality candidates, 4 of them (CrO_3_Y, Eu_2_GeSe_4_, Cl_3_CoTl, and MnO_3_Y) had metallic endpoints, 2 candidates (Cl_3_CrRb and Br_3_MnRb) had metallic interpolation structures, and 2 candidates (Cl_4_CoRb_2_ and B_7_ClCr_3_O_13_) did not have smooth energy trends due to fluctuating band gaps and magnetic moments in the interpolations. We note that most of these 8 candidates are non-*d*^0^ systems, and therefore expected to exhibit magnetic order. This suggests that the primary impact to the robustness of our workflow is the level of DFT used and the magnetic orderings considered; since PBE tends to underestimate band gaps, and since ferromagnetically-ordered systems tend to be overwhelmingly metallic, several of these candidates are experimentally insulating but are metallic in our workflow.

### Comparison to experimental measurements in Landolt-Börnstein series

To validate that our workflow calculates polarizations that can be used to guide experimental efforts, we compare to tabulated experimentally measured polarizations of known ferroelectrics in Landolt-Börnstein - Group III Condensed Matter - Ferroelectrics and Related Substances^19–23^. This series classifies hundreds of ferroelectrics into a 72 class numbering scheme. We note that polarization values for ferroelectrics in the Landolt-Börnstein series volumes 36A, 36B and 36C may be superseded by more recent experimental measurements. Experimentally measured polarizations depend greatly on the quality of the sample and the method used. For many ferroelectrics, polarization measurements made across decades vary greatly depending on these factors. Any comparison between theory must be taken in this context.

**Fig. 9:**
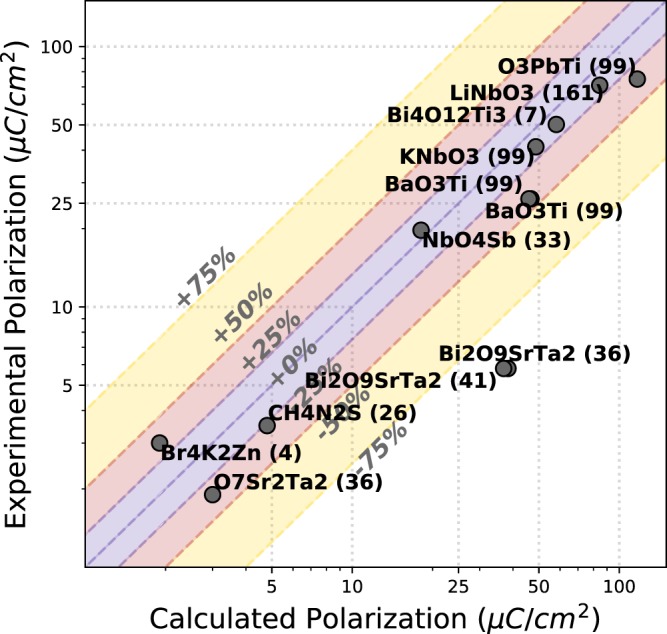
Log-log plot of experimental vs. calculated polarization for ferroelectric materials in the Landolt-Börnstein series with polarization value larger than 1 *μC*/*cm*^2^. 6 materials with polarization value equal to or smaller than 1 *μC*/*cm*^2^ are not shown in plot but are included in the Table 7. Colored regions show experimental values within ±25%, ±50%, and ±75% of calculated values.

Nonetheless, plots of polarizations values calculated by our workflow vs. experimental spontaneous polarizations reported in the Landolt-Börnstein series volumes 36A, 36B and 36C are shown in Fig. 9 and tabulated in Table 7. We only compare search candidates to Landolt-Börnstein entries that match in composition and polar space group. For example, NaNbO_3_ is LB Number 1A-1 and has a polarization of 12 *μC*/*cm*^2^ for the 161 space group structure. However, all the NaNbO_3_ found in our search are orthorhombic (space groups 26 and 29), so we do not make the comparison.

**Table 7:** Table of calculated versus experimentally measured polarizations as given in the Landolt-Börnstein - Group III: Condensed Matter - Ferroelectrics and Related Substances^19–21^.

Search ID	Formula	Polarization (*μC*/*cm*^2^)	LB number	Exp.	Exp. Ref.
51	B_7_ClMg_3_O_13_ (29)	0.3	18A-1	0.08	^92^
68	BaO_3_Ti (38)	49.9	1A-10	Not available	^93^
69, 70	BaO_3_Ti (99)	46.7, 45.9	1A-10	26	^93^
71	BaO_3_Ti (160)	49.9	1A-10	Not available	^93^
79	Bi_2_Nb_2_O_9_Pb (36)	38.1	9A-10	Not available	^94^
81	Bi_2_O_9_SrTa_2_ (41)	38.3	9A-12	5.8	^95^
80	Bi_2_O_9_SrTa_2_ (36)	36.9	9A-12	5.8	^95^
82	Bi_4_O_12_Ti_3_ (7)	58.1	9A-15	50.2	^96^
100	Br_4_K_2_Zn (4)	1.9	39A-16	3.0	^97^
111	CH_4_N_2_S (26)	4.8	50A-1	3.5	^98^
146	CdO_3_Ti (26)	37.2	1A-9	Debated	^99^
147	CdO_3_Ti (33)	34.8	1A-9	Debated	^99^
164, 165	Cl_4_K_2_Zn (33)	2.2, 0.5	39A-9	0.13	^100^
166	Cl_4_Rb_2_Zn (33)	0.5	39A-10	0.16	^101^
292	H_8_N_2_O_4_S (33)	0.0	39A-1	0.6 max at −51.5 °C, reversal by 0.05 approaching −260 °C	^102^
302	HfO_3_Pb (32)	19.8	1A-16	Antiferroelectric (62).	^103^
309	K_2_O_4_Se (33)	0.7	39A-2	0.15	^104^
313	KNbO_3_ (38)	51.3	1A-2	Not available	^105^
314	KNbO_3_ (99)	48.7	1A-2	41.2	^105^
315	KNbO_3_ (160)	50.7	1A-2	Not available	^105^
331	LiNbO_3_ (161)	84.5	2A-1	71	^106,107^
365	NbO_4_Sb (33)	18.1	5A-2	19.7	^108^
389	O_3_PbTi (99)	116.8	1A-11	75	^109,110^
390	O_3_PbZr (32)	23.5	1A-15	16 on cooling. Antiferroelectric (55).	^111^
404	O_5_PTiTl (33)	22.1	35A-13	Not available	^112^
407	O_7_Sr_2_Ta_2_ (36)	3.0	8A-6	1.9	^113^

“Not available” in the Experimental Reference column (Exp. Ref.) indicates a polarization value was not provided in these references. We make comparisons only between search candidates that have a polar space group compatible with the Landolt-Börnstein reference. The search ID being a simplified unique identifier, defined for the purposes of our work, for pairs of structures used in our workflow search.

For experimental polarizations greater than 10 *μC*/*cm*^2^, the majority of experimental polarizations are between 25% and −50% of those that we calculate, well within an order of magnitude. The exceptions are both polar structures of Bi_2_O_9_SrTa_2_ (space groups 36 and 41), which are calculated to have polarizations much greater than their experimental values. Multiple entries of a given formula indicate multiple calculations for different structure pairs in our dataset for the same compound. Compounds with polarizations of less than or equal to 1 *μC*/*cm*^2^ are not shown on the plot given the log-log scale. For polarizations less than 5 *μC*/*cm*^2^, we see our calculations capture the general trends of the experimental polarizations.

We find that the PBE functional we use for our DFT calculations overestimates polarizations. This is partially due to unit cells relaxed with PBE having larger than experimental volumes and thus larger distortions.

## Usage Notes

In this work, we present 413 nonpolar-polar structure pairs in the Materials Project database that are compatible with a second-order phase transition as ferroelectric candidates and perform DFT calculations of total energy, band gap, and polarization for these structures pairs.

This dataset offers the first opportunity to compare a large number of known, previously proposed, and new ferroelectrics side by side with the same methodology. We believe by setting strict criteria for ferroelectricity and casting a wide-net using high-throughput searches, we will find candidates that challenge and advance our understanding of ferroelectric phenomena. As seen in our candidates, there may be ferroelectrics waiting to be discovered that defy our expectations. This dataset will be useful for creating new tools and criteria for analyzing diverse ferroelectrics.

The infrastructure provided by the Bilbao Crystallographic Server, FireWorks, pymatgen, and atomate is crucial to being able to perform these types of searches efficiently. Thus, we also provide our code and data for these searches with the hope they will provide access for others to perform and develop similar searches.

Our code for performing structural interpolations and polarization calculations has been incorporated into the pymatgen and atomate packages. We also provide the code that we use to create the interface that we used to view our candidates in aggregate.

The workflow we have presented can be extended to any crystal structure database, experimental and hypothetical. Several modifications can be made to this workflow to extend the scope of these searches. Notably, extending our workflow to treat different magnetic orderings will enable it to treat multiferroics. Additionally, the same DFT workflow can be used to screen any experimentally measured polar structure - even one without an existing nonpolar reference - by generating nonpolar reference structures with BCS Pseudosymmetry^7^. Our workflow can also be adapted to perform species substitutions and find symmetry relations between structure types, classes of structures that share space groups, Wyckoff positions, and other lattice similarities.

## Data Availability

VASP version 5.3.5 used to perform DFT calculations is a proprietary code. The Bilbao Crystallographic Server (BCS) is freely available on-line at http://www.cryst.ehu.es. Fireworks, atomate, and pymatgen are python packages accessible on GitHub. Fireworks and atomate are released under a modified Berkeley Software Distribution (BSD) License. pymatgen is released under a Massachusetts Institute of Technology (MIT) License. Both MIT and BSD licenses are open-source and permit both commercial and non-commercial use. Our workflow code is included since atomate version 0.6.7 and our analysis code is available in pymatgen since v2019.2.4. We also use the following python packages in our analysis and Graphical Representation of Results: numpy, scipy, matplotlib, ipython, and jupyter^80,82–86^. These packages are freely available through the Python Package Index (https://pypi.org/). Our code for recovering the same branch polarization from polarization calculations has been contributed to pymatgen under the pymatgen.analysis.ferroelectricity module. Our code for the DFT and polarization analysis workflows for performing polarization calculations has been contributed to atomate under the atomate.vasp.workflows.base.ferroelectric module. We also provide code for the interface that we used to view our candidates in aggregate. The web interface for the current work is hosted at http://blondegeek.github.io/ferroelectric_search_site/. The code for the interface can be found at http://github.com/blondegeek/ferroelectric_search_site.

## Online-only Tables

**Online-only Table 1:** Known and proposed high quality ferroelectric candidates and their space groups, polarizations and band gaps.

Formula	Polar Space Group	Nonpolar Space Group	Polarization (μ C/cm^2)	Band Gap (eV)	Search ID	Subcategory
Ag_3_IS	4	155	0.7	0.3	11	Proposed by theory^114^
		221	0.7	0.3	12	
	146	155	11.7	0.5	13	
Al_2_BaO_4_	173	182	0.3	4.1	18	Proposed by theory^115^
	173	182	0.3	4.1	19	
Al_2_CaH_4_O_10_Si_2_	33	63	4.2	5.1	20	
Al_3_F_19_Pb_5_	108	140	13.9	5.2	21	
AlBiO_3_	161	221	80.3	3.1	23	Perovskite
	185	194	4.8	0.8	24	
AlF_7_MgNa_2_	46	74	1.1	6.7	30	
B_2_K_3_Nb_3_O_12_	26	189	0.1	2.4	46	
B_3_CaH_5_O_8_	4	14	0.6	5.6	48	
B_7_ClMg_3_O_13_	29	219	0.3	5.7	51	Boracite
BaNiO_3_	186	194	3.7	1.5	66	
BaO_3_Ti	38	221	49.9	2.4	68	Perovskite
	99	221	46.7	1.8	69	
			45.9	1.8	70	
	160	221	49.9	2.5	71	
BaO_5_Ti_2_	5	12	15.3	2.2	72	
BeF_4_H_8_N_2_	33	62	0.6	6.6	75	(NH4)2SO4 family
			0.6	6.6	76	
Bi_2_Nb_2_O_9_Pb	36	139	38.1	2.3	79	Perovskite
Bi_2_O_9_SrTa_2_	36	139	36.9	2.5	80	Perovskite
	41	139	38.3	2.3	81	
Bi_4_O_12_Ti_3_	7	139	58.1	2.4	82	
	41	139	17	2.4	83	
			11.5	1.6	84	
BiCl_8_F_4_H_3_K_6_	186	194	0.1	4.2	85	
BiFeO_3_†	160	221	124.5	1.8	89	Perovskite
BiInO_3_	33	62	64.7	2.8	91	Perovskite
		221	64.7	2.8	92	
BiO_3_Sc	9	15	5.7	2.7	94	Hexagonal manganite-like
	9	221	5.7	2.7	95	
Br_4_K_2_Zn	4	11	1.9	3.7	100	(NH4)2SO4 family
C_3_ClH_10_NO_4_	4	11	0.3	5.6	108	Proposed by theory^116^
CdO_3_Ti	26	62	37.2	2.5	146	Perovskite
	33	62	34.8	2.4	147	
Cl_3_CsPb	38	221	2.3	2.6	158	Perovskite
Cl_4_K_2_Zn	33	62	2.2	4.4	164	(NH4)2SO_4_ family
			0.5	4.5	165	
Cl_4_Rb_2_Zn	33	62	0.5	4.5	166	(NH4)2SO_4_ family
			0.3	4.4	167	
ClH	44	139	57.4	5.2	171	
CsF_3_Pb	161	221	0.7	3.4	195	Perovskite; Proposed by Theory^117^
CsO_4_PZn	33	62	1	4	196	
F_4_MgSr	4	11	10.7	6.7	220	Proposed by theory^118^
H_2_KO_4_P	9	14	0.5	5.2	256	
	43	122	5.2	5.4	257	
H_2_O	4	19	13.5	5.5	264	
			13.5	5.5	265	
H_2_O_4_PRb	43	122	5.4	5.2	271	Hydroxyl
H_8_N_2_O_4_S	33	62	0	5.1	292	(NH4)2SO_4_ family
HfO_2_	29	225	53.4	4.4	301	
HfO_3_Pb	32	55	19.8	2.8	302	Perovskite
HfO_3_Sr	99	221	15.1	3.8	303	Perovskite
K_2_O_4_Se	33	62	0.7	3.7	309	(NH4)2SO_4_ family
KNbO_3_	38	221	51.3	2.1	313	Perovskite
	99	221	48.7	1.6	314	
	160	221	50.7	2.3	315	
LiNbO_3_	161	167	84.5	3.4	331	Perovskite
MgO_3_Si	33	60	1.1	4.7	346	Perovskite
N_6_Pb	33	62	0.4	2.3	358	
NaNbO_3_	26	127	53.9	2.4	360	Perovskite
	29	57	31.5	2.4	361	
		63	31.5	2.4	362	
NbO_4_Sb	33	52	18.1	2.4	365	
O_2_Zr	29	225	51.2	3.7	388	
O_3_PbTi	99	221	116.8	2	389	Perovskite
O_3_PbZr	32	55	23.5	2.8	390	Perovskite
O_3_W	185	191	21	1.5	399	
			21	1.5	400	
		193	21	1.5	401	
O_5_PTiTl	33	52	22.1	2.7	404	
O_7_Sr_2_Ta_2_	36	63	3	2.9	407	Perovskite
			124.5			

Known candidates are in light gray. All candidates are non-magnetic unless indicated with a † which indicates the candidates are calculated to be ferromagnetic. Only three of all high-quality (know, proposed, and new) candidates contain atoms with nonzero magnetic moments: BiFeO_3_ (Search ID 89), CrP_3_O_9_ (Search ID 191), LiO_4_PV (Search ID 342). Please see text for discussion of workflow performance on magnetic properties. The search ID being a simplified unique identifier, defined for the purposes of our work, for pairs of structures used in our workflow search. Spaces in table implies the same space group number as the previous row.

**Online-only Table 2:** New high quality ferroelectric candidates and their space groups, polarizations and band gaps.

Formula	Polar Space Group	Nonpolar Space Group	Polarization (μ C/cm^2)	Band Gap (eV)	Search ID	Subcategory
Ag_10_Br_3_Te_4_	36	63	3.9	0.9	1	
Ag_2_O_4_W	34	58	1	1.8	4	
Ag_2_S	4	11	21.1	0.4	5	
		64	17.6	0.4	7	
	36	63	0.4	1.4	9	
AgAl_11_O_17_	40	63	2.4	3.1	15	
AgC_2_O_2_	5	15	0	1	17	
AlCH_2_NaO_5_	46	74	0.8	5	25	
AlCl_4_Hg_2_Sb	33	60	0.5	0.9	26	
AlH_3_O_3_	1	2	0.1	5	32	Hydroxyl
	7	61	8.1	5	33	
AlN	186	194	133	4.1	38	
AuCl_4_K	7	14	0.3	1.4	43	
B_13_C_2_Li	44	74	0.8	2.6	45	
B_2_O_6_Zn_3_	9	15	0.1	2.7	47	
Ba_2_F_7_Y	4	14	0.7	6.5	57	
BaC_2_CaO_6_	4	11	0	4.8	59	
BaCO_3_	4	11	1	4.5	60	Carbon-oxygen compound
BaCl_5_La	4	62	26.1	4	61	
BiCuO_8_W_2_	1	2	0.8	1.3	86	
BiO_3_Y	185	194	9.7	2.1	96	Hexagonal manganite-like
Br_3_CsGe	8	221	19.3	1.7	97	
C_2_HO_2_	6	11	3.4	2.8	106	Carbon-oxygen compound
C_2_HgN_2_S_2_	5	12	0.9	2.1	107	
C_6_Cu_2_H_10_N_4_S_3_	4	14	0	2.8	109	
CCs_4_O_4_	146	215	1.7	1.6	110	Carbon-oxygen compound
CH_4_N_2_S	26	62	4.8	3.3	111	
CHO_2_Tl	33	52	0.8	3.5	112	Carbon-oxygen compound
CK_4_O_4_	5	121	16.7	2	113	Carbon-oxygen compound
			16.6	2	114	
	8	121	10.2	2.2	115	
	146	215	2.6	2.3	116	
	160	215	14.6	1.9	117	
CLi_4_O_4_	5	121	49	5	118	Carbon-oxygen compound
			49	5	119	
			0.1	4.8	120	
	160	215	16.6	4.2	121	
CN_2_Pb	33	62	10.5	1.9	122	
CNa_4_O_4_	5	121	39.2	2.2	123	Carbon-oxygen compound
			39.2	2.2	124	
	8	215	12.6	1.7	125	
	146	215	3.6	1.9	126	
			56.2	2.2	127	
CO_4_Rb_4_	5	23	14.6	1.4	128	Carbon-oxygen compound
		121	14.6	1.4	129	
			14.6	1.4	130	
	146	215	0.6	1.7	131	
	160	215	42.8	0.4	132	
Ca_5_ClO_12_P_3_	173	176	2.1	5.4	138	
CaF_2_	26	123	16.5	6.3	139	Fluoride
Cd_2_ClP_3_	9	15	0.8	1.3	144	
CdCl_2_	5	12	48.4	3.1	145	
Cl_4_GaHg_2_Sb	33	60	0.5	0.8	162	
ClH_3_O_5_	33	62	5.2	5.4	172	
ClH_4_NO_4_	33	62	2.2	5.3	173	
ClIn	36	63	3.8	1.4	174	
CrO_3_	33	62	0.4	1.6	188	
CrO_9_P_3_†	9	15	0.3	3.2	191	
Cs_2_HgI_4_	4	11	0.3	2.1	192	
Cs_2_N_2_Tb_6_Te_7_	8	12	0.2	0.8	193	
Cs_2_O_3_Pb	36	63	7.2	1.4	194	
CuI	7	216	0	1.2	204	
Er_2_F_7_K	33	62	1.5	6.9	210	Fluoride
F_2_HRb	46	140	1.6	6.7	212	Fluoride
F_2_Pb	41	225	5	4.4	213	Fluoride
F_3_Nd	185	193	17.6	7.7	214	Fluoride
F_3_PbRb	9	221	4	3.8	215	Perovskite
F_5_V	9	15	1	2.8	223	Fluoride
			29.7	3.3	224	
F_6_LiV	33	225	1.3	3.2	225	Fluoride
FTl	39	225	6.4	3.1	229	Fluoride
GaLuO_3_	185	194	8	2.9	249	Hexagonal manganite-like
GaN_5_O_14_	5	82	0	1.3	252	
GaO_3_Sc	185	194	10	3.2	253	Hexagonal manganite-like
H_2_Mg	29	60	30.9	2.4	258	
H_2_MoO_4_	1	2	0.2	3.2	260	
			39.2	2.5	261	
			16.3	2.1	262	
H_2_O_10_U_3_	1	2	23	1.8	266	
			23	1.8	267	
H_2_O_2_Sr	26	62	1.2	4	268	Hydroxyl
			1.2	4	269	
H_2_O_4_S	5	15	20.4	6.1	272	Hydroxyl
	9	15	51	5.8	273	
H_2_O_4_Sn_3_	9	114	1.6	2.5	275	Hydroxyl
H_2_O_4_U	36	64	2.9	1.9	277	Hydroxyl
			2.9	1.9	278	
H_3_LaO_3_	173	176	17.9	4	279	Hydroxyl
H_3_O_3_Pr	6	11	0.1	3.8	280	Hydroxyl
		176	0.1	3.8	281	
H_3_O_3_Y	173	176	7.7	3.9	282	Hydroxyl
H_3_O_4_P	7	14	4.1	5.7	283	
			4.1	5.7	284	
H_4_O_5_S	7	14	1	5.7	286	
H_5_IO_6_	7	14	0.3	2.3	289	
HInO_2_	31	58	13.2	1.8	293	Hydroxyl
HK_2_NO_6_S_2_	9	15	3.2	5.3	294	
HNaO	4	11	10.7	3	295	Hydroxyl
		63	10.7	3	296	
HORb	4	11	10.5	3.4	300	Hydroxyl
ILi_6_PS_5_	9	216	2.6	2.3	304	
K_2_O_7_Zn_6_	102	136	2.5	0.8	310	
K_3_S_4_Sb	36	217	1	2.2	311	
KLaS_4_Si	4	11	7.4	2.8	312	
LiO_12_P_3_Zr_2_	5	167	8	4.2	341	
LiO_4_PV^†^	33	60	0.1	2.7	342	
MgN_2_O_6_	29	205	0.7	3.5	345	
MoO_3_	7	62	4.7	1.9	353	
N_2_O_6_Zn	29	205	1.1	3.3	356	
N_3_Na	5	12	51.8	4.1	357	
NbO_5_P	33	62	4	2.3	366	
O_23_Rb_6_Si_10_	38	189	0.1	4.3	370	
			0.5	4.2	371	
O_2_Si	4	20	0	5.4	373	
		182	0	5.4	374	
			0.4	5.6	375	
	8	12	0	5.5	378	
	9	194	0.1	5.5	379	
	36	63	0.2	5.7	383	
			0	5.6	384	
		64	0.2	5.4	385	
	44	119	0	5.7	386	
O_3_SbY	185	194	7.5	1.5	391	Hexagonal manganite-like
O_3_ScY	185	194	6.6	3.1	392	Hexagonal manganite-like
O_3_Te	185	194	0	0.5	394	
OPb	29	57	54.3	2.2	409	

All candidates are non-magnetic unless indicated with a † which indicates the candidates are calculated to be ferromagnetic. Only three of candidates contain atoms with nonzero magnetic moments: BiFeO_3_ (Search ID 89), CrP_3_O_9_ (Search ID 191), LiO_4_PV (Search ID 342). Please see text for discussion of workflow performance on magnetic properties. The search ID being a simplified unique identifier, defined for the purposes of our work, for pairs of structures used in our workflow search. Spaces in table implies the same space group number as the previous row.

**Online-only Table 3:** Search ID, alphabetical formula, Materials Project ID and Workflow IDs for high quality candidates.

Search ID	Alphabetical Formula as formatted in Materials Project database	Polar MP ID	Nonpolar MP ID	Workflow ID
1	Ag10Br3Te4	mp-568386	mp-568392	wfid_1562345081.92745142
2	Ag2BiO3	mp-561113	mp-558712	wfid_1562345066.59186560
3	Ag2BiO3	mp-23558	mp-558712	wfid_1562345080.63153360
4	Ag2O4W	mp-637188	mp-504466	wfid_1562345080.72611869
5	Ag2S	mp-560025	mp-556225	wfid_1562345112.11438196
6	Ag2S	mp-560025	mp-36216	wfid_1562345112.31901092
7	Ag2S	mp-560025	mp-32791	wfid_1562345112.28048731
8	Ag2S	mp-38511	mp-32791	wfid_1562345109.86187522
9	Ag2S	mp-32669	mp-36216	wfid_1562345080.93250415
10	Ag2S	mp-32669	mp-32791	wfid_1562345081.72797057
11	Ag3IS	mp-675879	mp-676561	wfid_1562345089.24703674
12	Ag3IS	mp-675879	mp-558189	wfid_1562345097.63538177
13	Ag3IS	mp-22995	mp-676561	wfid_1562345085.94165367
14	Ag3IS	mp-22995	mp-558189	wfid_1562345089.76230197
15	AgAl11O17	mp-849760	mp-766293	wfid_1562345083.68955336
16	AgAlO3	mvc-3476	mvc-15935	wfid_1562345112.91840254
17	AgC2O2	mp-600237	mp-654937	wfid_1562345064.01802743
18	Al2BaO4	mp-4202	mp-619456	wfid_1562345097.07894293
19	Al2BaO4	mp-4202	mp-3828	wfid_1562345097.68976538
20	Al2CaH4O10Si2	mp-24603	mp-733653	wfid_1562345079.80563577
21	Al3F19Pb5	mp-541732	mp-557911	wfid_1562345086.15212454
22	Al4La	mp-571423	mp-21109	wfid_1562345105.73961799
23	AlBiO3	mp-551918	mp-23080	wfid_1562345104.79543572
24	AlBiO3	mvc-3494	mvc-13972	wfid_1562345095.70366936
25	AlCH2NaO5	mp-699136	mp-644506	wfid_1562345113.04187974
26	AlCl4Hg2Sb	mp-570828	mp-568001	wfid_1562345074.60128275
27	AlCoO3	mvc-3774	mvc-13532	wfid_1562345095.84764302
28	AlCrO3	mvc-3775	mvc-13996	wfid_1562345097.53150367
29	AlCuO3	mvc-3501	mvc-15945	wfid_1562345104.05261108
30	AlF7MgNa2	mp-19931	mp-6319	wfid_1562345084.84452891
31	AlH3O3	mp-625318	mp-625316	wfid_1562345113.85119430
32	AlH3O3	mp-626435	mp-626414	wfid_1562199295.69225680
33	AlH3O3	mp-626587	mp-626605	wfid_1562345066.90756711
34	AlMnO3	mvc-3777	mvc-15992	wfid_1562345104.31488404
35	AlMo4S8	mp-554868	mp-3861	wfid_1562345097.39878133
36	AlMo4S8	mvc-16083	mp-3861	wfid_1562345099.89947666
37	AlMoO3	mvc-3779	mvc-14006	wfid_1562345104.48448591
38	AlN	mp-661	mp-13178	wfid_1562345088.40370432
39	AlNiO3	mvc-3776	mvc-14043	wfid_1562345104.16630482
40	AlO3Ti	mvc-3466	mvc-13964	wfid_1562345113.24875150
41	As2NiTm2	mp-568266	mp-11581	wfid_1562345088.19078870
42	AsNi	mp-590	mp-2346	wfid_1562345096.13686232
43	AuCl4K	mp-27181	mp-568986	wfid_1562345066.70855532
44	AuTe2	mp-571547	mp-567525	wfid_1562345104.42703805
45	B13C2Li	mp-638070	mp-655591	wfid_1562345084.34740380
46	B2K3Nb3O12	mp-557711	mp-15248	wfid_1562345108.76820757
47	B2O6Zn3	mp-559949	mp-542833	wfid_1562345069.20163833
48	B3CaH5O8	mp-560899	mp-705495	wfid_1562345061.85187620
49	B7BrMn3O13	mp-579836	mp-567153	wfid_1562345110.99651178
50	B7ClCr3O13	mp-579770	mp-566691	wfid_1562345108.33484522
51	B7ClMg3O13	mp-23087	mp-23617	wfid_1562345107.17640773
52	B7IMn3O13	mp-31917	mp-565322	wfid_1562345099.25830051
53	BC2N	mp-629458	mp-1008523	wfid_1562345103.49034383
54	BCoLi2O4	mp-761299	mp-771049	wfid_1562345117.22594957
55	Ba2Co4Nd2O11	mp-561781	mp-24879	wfid_1562345070.07733415
56	Ba2CrO4	mp-566511	mp-19703	wfid_1562345076.48947736
57	Ba2F7Y	mp-768350	mp-777744	wfid_1562345060.94342955
58	Ba3C5Ce2F2O15	mp-667381	mp-581090	wfid_1562199296.78790598
59	BaC2CaO6	mp-644852	mp-6568	wfid_1562199296.60378780
60	BaCO3	mp-762225	mp-34195	wfid_1562199296.73456920
61	BaCl5La	mp-770427	mp-770125	wfid_1562345112.71860945
62	BaEuFe2O5	mp-639347	mp-656144	wfid_1562345085.29703104
63	BaFe2S4	mp-675078	mp-27660	wfid_1562345085.09598476
64	BaFe2S4	mp-675078	mp-676036	wfid_1562345085.23366390
65	BaMnO3	mp-19267	mp-19156	wfid_1562345090.52467225
66	BaNiO3	mp-19241	mp-19138	wfid_1562345089.13746210
67	BaO3Ti	mp-995191	mp-19990	wfid_1562345103.16533381
68	BaO3Ti	mp-5777	mp-2998	wfid_1562345097.33024928
69	BaO3Ti	mp-12992	mp-2998	wfid_1562345085.34572722
70	BaO3Ti	mp-5986	mp-2998	wfid_1562345085.18772603
71	BaO3Ti	mp-5020	mp-2998	wfid_1562345096.05165490
72	BaO5Ti2	mp-555966	mp-3943	wfid_1562345063.80485743
73	BaS3V	mp-3451	mp-4227	wfid_1562345096.68042421
74	BaSe3V	mp-676597	mp-27363	wfid_1562345111.62539924
75	BeF4H8N2	mp-24614	mp-604245	wfid_1562345113.37855627
76	BeF4H8N2	mp-24614	mp-720982	wfid_1562345114.26291952
77	Bi2MoO6	mp-25708	mp-567075	wfid_1562345072.07030301
78	Bi2MoO6	mp-25708	mp-567326	wfid_1562345072.66801127
79	Bi2Nb2O9Pb	mp-583454	mp-23101	wfid_1562345096.76465275
80	Bi2O9SrTa2	mp-23089	mp-554675	wfid_1562345092.91429073
81	Bi2O9SrTa2	mp-559951	mp-554675	wfid_1562345093.12462394
82	Bi4O12Ti3	mp-723064	mp-23335	wfid_1562345067.21007940
83	Bi4O12Ti3	mp-23427	mp-23335	wfid_1562345115.68239992
84	Bi4O12Ti3	mp-622198	mp-23335	wfid_1562345090.67222161
85	BiCl8F4H3K6	mp-696998	mp-723540	wfid_1562345088.64692719
86	BiCuO8W2	mp-565192	mp-615173	wfid_1562199296.16002539
87	BiCuYb	mp-22953	mp-22960	wfid_1562345089.00815378
88	BiFeO3	mp-601706	mp-561388	wfid_1562345117.60950601
89	BiFeO3	mp-24942	mp-561388	wfid_1562345068.96362730
90	BiFeO3	mp-24932	mp-561388	wfid_1562345095.33810901
91	BiInO3	mp-556892	mp-561102	wfid_1562345078.25607094
92	BiInO3	mp-556892	mp-545379	wfid_1562345104.93967568
93	BiO2	mvc-9645	mp-32548	wfid_1562345101.07858072
94	BiO3Sc	mp-555313	mp-555769	wfid_1562345069.03136189
95	BiO3Sc	mp-555313	mp-550008	wfid_1562345104.65432837
96	BiO3Y	mvc-3479	mvc-15941	wfid_1562345100.97005996
97	Br3CsGe	mp-642739	mp-570223	wfid_1562345114.09410592
98	Br3MnRb	mp-568231	mp-29763	wfid_1562345090.03425890
99	Br3RbV	mp-29314	mp-570099	wfid_1562345089.82930048
100	Br4K2Zn	mp-23535	mp-23495	wfid_1562199296.55106864
101	BrH	mp-632229	mp-634105	wfid_1562345082.80546132
102	BrH	mp-632229	mp-23903	wfid_1562345083.08491158
103	BrMnSbSe2	mp-639335	mp-655834	wfid_1562345065.32859164
104	C2CoLi2O6	mp-765126	mp-763828	wfid_1562345060.79723600
105	C2CoLi2O6	mp-765128	mp-763828	wfid_1562345060.64209065
106	C2HO2	mp-675395	mp-23680	wfid_1562345111.73469268
107	C2HgN2S2	mp-610992	mp-655275	wfid_1562345063.29510504
108	C3ClH10NO4	mp-554570	mp-560498	wfid_1562199296.21214937
109	C6Cu2H10N4S3	mp-600236	mp-555729	wfid_1562345061.28565377
110	CCs4O4	mp-562815	mp-605824	wfid_1562345090.37034900
111	CH4N2S	mp-721896	mp-23930	wfid_1562345071.44504562
112	CHO2Tl	mp-558579	mp-557687	wfid_1562345073.70348192
113	CK4O4	mp-551561	mp-549687	wfid_1562345114.85602856
114	CK4O4	mp-551561	mp-549869	wfid_1562345064.72820430
115	CK4O4	mp-605843	mp-549869	wfid_1562345067.39360264
116	CK4O4	mp-545387	mp-551736	wfid_1562345092.46694443
117	CK4O4	mp-545630	mp-551736	wfid_1562345091.11202201
118	CLi4O4	mp-550320	mp-551740	wfid_1562345109.75844212
119	CLi4O4	mp-550320	mp-551848	wfid_1562345064.64947472
120	CLi4O4	mp-550498	mp-551740	wfid_1562345095.95394592
121	CLi4O4	mp-550593	mp-546202	wfid_1562345093.97216898
122	CN2Pb	mp-619032	mp-19727	wfid_1562345077.98674939
123	CNa4O4	mp-13274	mp-546551	wfid_1562345064.95566356
124	CNa4O4	mp-13274	mp-546707	wfid_1562345116.92380413
125	CNa4O4	mp-645295	mp-551886	wfid_1562345106.21088335
126	CNa4O4	mp-552623	mp-551886	wfid_1562345091.53870848
127	CNa4O4	mp-552941	mp-551886	wfid_1562345092.72215049
128	CO4Rb4	mp-551176	mp-550679	wfid_1562345106.35940545
129	CO4Rb4	mp-551176	mp-547898	wfid_1562345115.59588544
130	CO4Rb4	mp-551176	mp-545760	wfid_1562345065.02973794
131	CO4Rb4	mp-550314	mp-546320	wfid_1562345091.36271002
132	CO4Rb4	mp-551690	mp-546320	wfid_1562345111.91150344
133	Ca2FeO6W	mvc-12913	mp-619611	wfid_1562345117.46280857
134	Ca3Mn2O7	mp-19042	mp-19610	wfid_1562345081.50183207
135	Ca3Mn2O7	mvc-11576	mp-19610	wfid_1562345099.00022301
136	Ca3Mn2O7	mp-19042	mp-19124	wfid_1562345100.32022475
137	Ca3Mn2O7	mvc-11576	mp-19124	wfid_1562345098.61366590
138	Ca5ClO12P3	mp-39460	mp-554236	wfid_1562345087.78522502
139	CaF2	mp-560030	mp-554355	wfid_1562345100.02925969
140	CaFe2O4	mvc-12582	mvc-8188	wfid_1562345116.80707800
141	CaFe2O4	mvc-12583	mvc-8188	wfid_1562345117.00610059
142	CaFeO6W	mvc-10916	mvc-14934	wfid_1562345116.27690236
143	CaNi2O4	mvc-12644	mvc-7742	wfid_1562345116.53277296
144	Cd2ClP3	mp-29246	mp-644431	wfid_1562345068.11764927
145	CdCl2	mp-632403	mp-695850	wfid_1562345118.41939438
146	CdO3Ti	mp-20940	mp-14550	wfid_1562345071.77195440
147	CdO3Ti	mp-5052	mp-14550	wfid_1562345078.17038961
148	CdTe	mp-685146	mp-1008471	wfid_1562345102.26130693
149	CdTe	mp-685146	mp-2388	wfid_1562345082.98370782
150	CeCu4Sn	mp-640286	mp-655580	wfid_1562345115.39463102
151	CeCuSn	mp-22683	mp-22761	wfid_1562345088.97236356
152	CeKS4Si	mp-22809	mp-11170	wfid_1562199297.04974693
153	Cl2H8MgO12	mp-989229	mp-865188	wfid_1562345063.58842327
154	Cl3CoTl	mp-567430	mp-569753	wfid_1562345112.15249459
155	Cl3CrCs	mp-610955	mp-570326	wfid_1562345089.18755185
156	Cl3CrRb	mp-568864	mp-568887	wfid_1562345089.52001267
157	Cl3CrRb	mp-568864	mp-30027	wfid_1562345064.81349297
158	Cl3CsPb	mp-675524	mp-23037	wfid_1562345092.64050822
159	Cl3CuRb	mp-571305	mp-568857	wfid_1562345064.33652828
160	Cl3CuRb	mp-571305	mp-569526	wfid_1562345108.13574082
161	Cl4CoRb2	mp-571242	mp-23076	wfid_1562345075.90964892
162	Cl4GaHg2Sb	mp-568031	mp-571190	wfid_1562345074.87030107
163	Cl4K2Zn	mp-653633	mp-653454	wfid_1562345109.96773133
164	Cl4K2Zn	mp-618177	mp-653454	wfid_1562345075.60077746
165	Cl4K2Zn	mp-647575	mp-653454	wfid_1562345106.46698464
166	Cl4Rb2Zn	mp-568350	mp-608314	wfid_1562345106.82651466
167	Cl4Rb2Zn	mp-616185	mp-608314	wfid_1562345076.32521814
168	Cl8Na2Ti3	mp-569978	mp-29474	wfid_1562345086.01893685
169	ClH	mp-632326	mp-634101	wfid_1562345118.36325774
170	ClH	mp-632326	mp-23722	wfid_1562345083.03981015
171	ClH	mp-684609	mp-634101	wfid_1562345116.34885480
172	ClH3O5	mp-625175	mp-625148	wfid_1562345076.84195890
173	ClH4NO4	mp-698084	mp-706586	wfid_1562345077.01048358
174	ClIn	mp-571636	mp-571555	wfid_1562345080.19562594
175	CoLi2O4Si	mp-764958	mp-764956	wfid_1562345093.33885886
176	CoLiO4P	mp-761753	mp-18915	wfid_1562345062.80425054
177	CoLiO4P	mp-761753	mp-761979	wfid_1562345062.56056403
178	CoN	mvc-15478	mp-1009078	wfid_1562345115.48425255
179	CoO3Y	mvc-3765	mvc-3570	wfid_1562345101.77376431
180	CoO6Si2	mvc-12012	mvc-11366	wfid_1562345116.15943930
181	CoO6Si2	mvc-15005	mvc-11366	wfid_1562345116.64943193
182	Cr2S3	mp-849081	mp-555569	wfid_1562345085.83637119
183	CrI3Rb	mp-27442	mp-676553	wfid_1562345107.98049651
184	CrLi3O4	mp-770632	mp-777303	wfid_1562345073.19170804
185	CrLiO4P	mp-761391	mp-25507	wfid_1562345097.86901705
186	CrLiO4P	mp-761399	mp-761401	wfid_1562345079.39507654
187	CrO3	mvc-13134	mp-779986	wfid_1562345115.52822945
188	CrO3	mp-779941	mp-779986	wfid_1562345077.57898854
189	CrO3	mvc-13999	mvc-11097	wfid_1562345100.48228079
190	CrO3Y	mvc-3768	mvc-3569	wfid_1562345101.55871265
191	CrO9P3	mp-566776	mp-566761	wfid_1562345068.26681921
192	Cs2HgI4	mp-567594	mp-28421	wfid_1562199296.32626762
193	Cs2N2Tb6Te7	mp-646007	mp-655613	wfid_1562345117.74137700
194	Cs2O3Pb	mp-21521	mp-21283	wfid_1562345081.30847872
195	CsF3Pb	mp-20282	mp-5811	wfid_1562345093.87340147
196	CsO4PZn	mp-559752	mp-18673	wfid_1562345078.54041242
197	Cu2Se	mp-684653	mp-22297	wfid_1562345109.82599426
198	Cu3Mo2O9	mp-649957	mp-639719	wfid_1562345076.59705060
199	Cu4LiO12P3	mp-761193	mp-26741	wfid_1562345069.50145207
200	Cu8O	mp-704745	mp-31217	wfid_1562345083.27533748
201	CuF4Li2	mp-753171	mp-753123	wfid_1562345117.37566277
202	CuGeLi2	mp-676117	mp-35841	wfid_1562345108.71707409
203	CuGeYb	mp-567768	mp-5111	wfid_1562345089.04666899
204	CuI	mp-673245	mp-22895	wfid_1562199296.66891517
205	CuLiO4P	mp-769278	mp-758750	wfid_1562345103.23786212
206	CuLiO4P	mp-757209	mp-25449	wfid_1562345080.06677653
207	CuLiO4P	mp-752507	mp-758750	wfid_1562345079.49749416
208	CuN	mvc-13841	mp-1008922	wfid_1562345115.20318466
209	CuSbYb	mp-11701	mp-9439	wfid_1562345089.09864953
210	Er2F7K	mp-27925	mp-558238	wfid_1562345077.72055408
211	Eu2GeSe4	mp-629088	mp-505740	wfid_1562199296.49909393
212	F2HRb	mp-677103	mp-29764	wfid_1562345084.74762742
213	F2Pb	mp-685150	mp-315	wfid_1562345083.62247248
214	F3Nd	mp-18511	mp-18074	wfid_1562345088.07662185
215	F3PbRb	mp-674508	mp-21043	wfid_1562345118.45837326
216	F4FeLi	mp-777891	mp-778352	wfid_1562345073.27618989
217	F4FeLi2	mp-777471	mp-777588	wfid_1562345079.15751718
218	F4Li2OV	mp-764695	mp-780857	wfid_1562345081.19754088
219	F4LiV	mp-764895	mp-766952	wfid_1562345091.69966218
220	F4MgSr	mp-561022	mp-556290	wfid_1562345060.28720726
221	F5FeK2	mp-579331	mp-555882	wfid_1562345075.11835588
222	F5H2Li2OV	mp-868263	mp-770536	wfid_1562345112.83065051
223	F5V	mp-765140	mvc-14312	wfid_1562345115.11396023
224	F5V	mp-766786	mvc-14312	wfid_1562345115.26181400
225	F6LiV	mp-765122	mp-765966	wfid_1562345101.99111429
226	F7FeNa2Ni	mp-566483	mp-558817	wfid_1562345084.18504979
227	F7LiMn2	mp-765204	mp-763085	wfid_1562345061.66535445
228	FNbO2	mp-752467	mp-35171	wfid_1562345068.05041391
229	FTl	mp-558134	mp-2175	wfid_1562345083.57628883
230	Fe2Li3O12P3	mp-762728	mp-853256	wfid_1562199295.93469828
231	Fe2O4Zn	mvc-12661	mvc-15076	wfid_1562345116.41517559
232	Fe3O4	mp-612405	mp-715614	wfid_1562345066.80008603
233	Fe3O4	mp-715275	mp-541907	wfid_1562345071.34228519
234	Fe3O4	mp-715275	mp-18731	wfid_1562345071.67063249
235	Fe4LiO12P3	mp-762896	mp-540020	wfid_1562345069.75195107
236	Fe7S8	mp-850411	mp-685128	wfid_1562345061.12554914
237	FeHO2	mp-625314	mp-625269	wfid_1562345062.25044610
238	FeHO2	mp-625314	mp-605437	wfid_1562345062.47321464
239	FeHO2	mp-625268	mp-625233	wfid_1562345077.64486953
240	FeHO2	mp-625268	mp-605437	wfid_1562345078.08008077
241	FeHO2	mp-626102	mp-743660	wfid_1562345081.65293768
242	FeLi2O4Si	mp-764344	mp-762566	wfid_1562345092.81949641
243	FeLiO4P	mp-762593	mp-765913	wfid_1562345099.64867399
244	FeLiO4P	mp-766763	mp-765913	wfid_1562345079.60737271
245	FeLiO4Si	mp-766664	mp-762643	wfid_1562345118.01517942
246	FeO2	mvc-12125	mp-25519	wfid_1562345100.60371473
247	FeO3Sc	mp-771123	mp-769970	wfid_1562345089.93657805
248	FeO3Y	mvc-3751	mvc-3556	wfid_1562345097.97802299
249	GaLuO3	mp-768505	mp-755342	wfid_1562345094.09550293
250	GaMo4S8	mp-559694	mp-2885	wfid_1562345089.31591687
251	GaMo4Se8	mp-567394	mp-5584	wfid_1562345096.94387271
252	GaN5O14	mp-541950	mp-557954	wfid_1562345064.51602404
253	GaO3Sc	mp-769079	mp-754165	wfid_1562345093.59972440
254	Gd3O7Os	mp-567291	mp-16825	wfid_1562345112.36603552
255	Gd3O7Ru	mp-683963	mp-17237	wfid_1562345111.31901335
256	H2KO4P	mp-757909	mp-24262	wfid_1562345103.54083952
257	H2KO4P	mp-23959	mp-696752	wfid_1562345096.22998438
258	H2Mg	mp-569051	mp-23711	wfid_1562345071.87614530
259	H2Mg	mp-569051	mp-1008901	wfid_1562345095.28203208
260	H2MoO4	mp-625600	mp-626577	wfid_1562199295.62978167
261	H2MoO4	mp-626582	mp-626577	wfid_1562199296.07296394
262	H2MoO4	mp-626586	mp-626577	wfid_1562199295.77439317
263	H2NiO2	mp-626843	mp-626794	wfid_1562345090.47289757
264	H2O	mp-557082	mp-558958	wfid_1562345062.67599870
265	H2O	mp-557082	mp-558226	wfid_1562345062.31879317
266	H2O10U3	mp-626114	mp-626104	wfid_1562199295.82524035
267	H2O10U3	mp-626114	mp-626062	wfid_1562199295.87807235
268	H2O2Sr	mp-625184	mp-27425	wfid_1562345070.54182585
269	H2O2Sr	mp-625184	mp-625191	wfid_1562345070.86402364
270	H2O2Zn	mp-625857	mp-625830	wfid_1562345062.14719002
271	H2O4PRb	mp-23667	mp-642831	wfid_1562345095.48459868
272	H2O4S	mp-625475	mp-690733	wfid_1562345114.14736365
273	H2O4S	mp-625445	mp-690733	wfid_1562345068.84228879
274	H2O4S	mp-625474	mp-690733	wfid_1562345068.50494058
275	H2O4Sn3	mp-625789	mp-625541	wfid_1562345091.78627029
276	H2O4Te	mp-625526	mp-625513	wfid_1562345065.26430213
277	H2O4U	mp-626885	mp-626876	wfid_1562345081.78625138
278	H2O4U	mp-626885	mp-626864	wfid_1562345082.84911275
279	H3LaO3	mp-625733	mp-625394	wfid_1562345086.50809338
280	H3O3Pr	mp-625452	mp-626361	wfid_1562345065.11062718
281	H3O3Pr	mp-625452	mp-625447	wfid_1562345065.17991627
282	H3O3Y	mp-625677	mp-24076	wfid_1562345086.70080946
283	H3O4P	mp-626450	mp-626464	wfid_1562345066.41605572
284	H3O4P	mp-626450	mp-626449	wfid_1562345066.26534577
285	H3O4P	mp-626450	mp-23902	wfid_1562345065.88747031
286	H4O5S	mp-626448	mp-626109	wfid_1562345065.45762179
287	H4O6Sr	mp-625836	mp-625812	wfid_1562345067.79383899
288	H4O6Sr	mp-625871	mp-625821	wfid_1562345119.03613496
289	H5IO6	mp-625256	mp-625174	wfid_1562345066.04864776
290	H5IO6	mp-625256	mp-27773	wfid_1562345065.65066324
291	H5NO2	mp-625109	mp-625108	wfid_1562345113.93677372
292	H8N2O4S	mp-24468	mp-23876	wfid_1562345077.14704718
293	HInO2	mp-504535	mp-632711	wfid_1562345073.13297542
294	HK2NO6S2	mp-695383	mp-706912	wfid_1562345068.66194719
295	HNaO	mp-626000	mp-23940	wfid_1562199296.04072234
296	HNaO	mp-626000	mp-23891	wfid_1562345093.29957585
297	HNaO	mp-625996	mp-23891	wfid_1562345109.01493217
298	HO2V	mp-626791	mp-626787	wfid_1562345071.60128680
299	HO2V	mp-626791	mp-626796	wfid_1562345071.21174354
300	HORb	mp-626721	mp-643043	wfid_1562199296.70345448
301	HfO2	mp-685097	mp-550893	wfid_1562345073.07864623
302	HfO3Pb	mp-669414	mp-22734	wfid_1562345073.51183116
303	HfO3Sr	mp-13108	mp-4551	wfid_1562345085.42724040
304	ILi6PS5	mp-950995	mp-985582	wfid_1562345070.64579894
305	ILiO3	mp-22955	mp-545343	wfid_1562345088.90631590
306	ILiO3	mp-613442	mp-545343	wfid_1562345088.85588763
307	INaO3	mp-559252	mp-545825	wfid_1562345117.14457677
308	In3Mg	mp-973320	mp-973308	wfid_1562345085.05162970
309	K2O4Se	mp-557025	mp-5226	wfid_1562345101.21075848
310	K2O7Zn6	mp-559112	mp-540728	wfid_1562345085.70573284
311	K3S4Sb	mp-9781	mp-9911	wfid_1562345091.21940324
312	KLaS4Si	mp-861938	mp-12924	wfid_1562345060.20349732
313	KNbO3	mp-5246	mp-935811	wfid_1562345096.61294252
314	KNbO3	mp-4342	mp-935811	wfid_1562345085.39184383
315	KNbO3	mp-7375	mp-935811	wfid_1562345096.53537136
316	LaN3W	mp-989524	mp-989455	wfid_1562345105.24353733
317	Li2MnO6Si2	mp-764791	mp-767686	wfid_1562345116.00036545
318	Li3Mg	mp-976139	mp-976254	wfid_1562345063.43801573
319	LiMn4O12P3	mp-32021	mp-853253	wfid_1562345070.20398004
320	LiMnO2	mp-775531	mp-775236	wfid_1562345089.69436506
321	LiMnO4P	mp-690866	mp-868359	wfid_1562345112.01290767
322	LiMnO4P	mp-765846	mp-765871	wfid_1562345114.75068713
323	LiMnO4P	mp-761551	mp-868359	wfid_1562345078.67804385
324	LiMnO4P	mp-766735	mp-868359	wfid_1562345079.01940689
325	LiMnO4P	mp-780646	mp-18997	wfid_1562345078.43784085
326	LiMnO4P	mp-761562	mp-765871	wfid_1562345098.18604043
327	LiMnO4P	mp-766735	mp-31939	wfid_1562345102.64344909
328	LiMnO4P	mp-780646	mp-765871	wfid_1562345079.70386079
329	LiMnO4P	mp-867520	mp-765871	wfid_1562345080.29869855
330	LiMnO4Si	mp-762828	mp-780325	wfid_1562345105.43550629
331	LiNbO3	mp-3731	mp-552588	wfid_1562345086.57526556
332	LiNi4O12P3	mp-868339	mp-868378	wfid_1562345070.95947283
333	LiNiO4P	mp-763217	mp-25614	wfid_1562345062.93664125
334	LiNiO4P	mp-763217	mp-762173	wfid_1562345063.47200918
335	LiNiO4P	mp-763217	mp-761990	wfid_1562345063.14923175
336	LiNiO4P	mp-763061	mp-32324	wfid_1562345098.08585876
337	LiNiO4P	mp-766636	mp-761990	wfid_1562345102.75577260
338	LiNiO4P	mp-772673	mp-761990	wfid_1562345080.47287897
339	LiNiO4P	mp-868169	mp-32324	wfid_1562345105.85799320
340	LiO12P3W2	mp-763531	mp-763372	wfid_1562345098.77384901
341	LiO12P3Zr2	mp-681439	mp-541661	wfid_1562345094.48631430
342	LiO4PV	mp-765022	mp-761338	wfid_1562345076.06485889
343	LiO4PV	mp-765022	mp-32425	wfid_1562345102.34349984
344	LiO4SiV	mp-767103	mp-767620	wfid_1562345079.26097922
345	MgN2O6	mp-776410	mp-771046	wfid_1562345119.24225097
346	MgO3Si	mp-557803	mp-5026	wfid_1562345075.73274781
347	MgRb3	mp-974981	mp-974940	wfid_1562345084.61541787
348	MnN	mvc-13808	mp-1009130	wfid_1562345116.38073018
349	MnO3Y	mp-19385	mp-19227	wfid_1562345094.72739682
350	MnO3Y	mp-19385	mvc-11553	wfid_1562345094.87268641
351	MnO3Y	mvc-16316	mvc-11553	wfid_1562345101.66519989
352	MnO3Y	mvc-16316	mp-19227	wfid_1562345100.17508190
353	MoO3	mp-715584	mvc-12752	wfid_1562345118.94396078
354	MoO3	mvc-13534	mvc-11096	wfid_1562345105.14252211
355	MoO3Y	mvc-3769	mvc-3559	wfid_1562345101.88114837
356	N2O6Zn	mp-778973	mp-772617	wfid_1562345118.64838432
357	N3Na	mp-634410	mp-570538	wfid_1562345117.90645376
358	N6Pb	mp-620058	mp-667338	wfid_1562345078.78578590
359	NOs	mp-999317	mp-1009496	wfid_1562345106.31147985
360	NaNbO3	mp-4681	mp-4419	wfid_1562345096.42937982
361	NaNbO3	mp-558920	mp-3671	wfid_1562345071.93467738
362	NaNbO3	mp-558920	mp-559354	wfid_1562345072.89491942
363	NaO11V6	mp-567072	mp-510616	wfid_1562345107.63740338
364	NaO11V6	mp-25156	mp-510616	wfid_1562345088.44942336
365	NbO4Sb	mp-3491	mp-3612	wfid_1562345074.16515924
366	NbO5P	mp-556918	mp-5803	wfid_1562345078.33658421
367	NiO3Y	mvc-3773	mvc-14342	wfid_1562345102.14561677
368	O11PbV6	mp-619128	mp-25790	wfid_1562345088.26528020
369	O13V7	mp-715598	mp-556332	wfid_1562199296.38256579
370	O23Rb6Si10	mp-27376	mp-561189	wfid_1562345094.99343391
371	O23Rb6Si10	mp-555837	mp-561189	wfid_1562345094.24965045
372	O2Sb	mp-230	mp-560098	wfid_1562345073.78468145
373	O2Si	mp-555891	mp-7648	wfid_1562345105.66292123
374	O2Si	mp-555891	mp-559091	wfid_1562345063.38077530
375	O2Si	mp-972808	mp-559091	wfid_1562345063.09303946
376	O2Si	mp-553881	mp-10948	wfid_1562345104.59178493
377	O2Si	mp-553881	mp-10064	wfid_1562345067.15640753
378	O2Si	mp-557881	mp-558351	wfid_1562345067.50179482
379	O2Si	mp-555235	mp-7087	wfid_1562345099.15877093
380	O2Si	mp-554089	mp-10948	wfid_1562345074.54062829
381	O2Si	mp-554089	mp-7905	wfid_1562345080.24041294
382	O2Si	mp-554089	mp-10064	wfid_1562345080.41388803
383	O2Si	mp-16964	mp-560826	wfid_1562345082.41349074
384	O2Si	mp-556218	mp-560826	wfid_1562345083.35483635
385	O2Si	mp-557264	mp-559313	wfid_1562345083.13307328
386	O2Si	mp-554946	mp-644923	wfid_1562345086.82500871
387	O2V	mp-715553	mp-714880	wfid_1562345090.13953873
388	O2Zr	mp-556605	mp-1565	wfid_1562345072.21799631
389	O3PbTi	mp-20459	mp-19845	wfid_1562345085.47191419
390	O3PbZr	mp-647557	mp-542903	wfid_1562345073.88793953
391	O3SbY	mvc-3460	mvc-14740	wfid_1562345100.87211791
392	O3ScY	mp-769007	mp-768479	wfid_1562345093.42856192
393	O3SnY	mvc-3464	mvc-13971	wfid_1562345100.77015926
394	O3Te	mvc-14734	mvc-14413	wfid_1562345105.02231844
395	O3TiY	mvc-3431	mvc-13995	wfid_1562345100.06969004
396	O3V2	mp-553955	mp-715514	wfid_1562345067.95646032
397	O3V2	mp-553955	mp-714906	wfid_1562345118.53916482
398	O3VY	mvc-3770	mvc-13691	wfid_1562345103.93946099
399	O3W	mp-32662	mp-559175	wfid_1562345090.19555210
400	O3W	mp-32662	mp-715590	wfid_1562345090.28098999
401	O3W	mp-32662	mp-32777	wfid_1562345087.96868747
402	O3W	mvc-13988	mvc-11457	wfid_1562345101.46683505
403	O3WY	mvc-3772	mvc-15989	wfid_1562345103.36826836
404	O5PTiTl	mp-6706	mp-559607	wfid_1562345074.29488196
405	O7OsSm3	mp-555639	mp-5447	wfid_1562345109.33811762
406	O7RuSm3	mp-555525	mp-5779	wfid_1562345109.06349917
407	O7Sr2Ta2	mp-13664	mp-12286	wfid_1562345080.99369532
408	O8W3	mp-715557	mp-19066	wfid_1562345114.96113485
409	OPb	mp-550714	mp-20878	wfid_1562345071.55443872
410	PSn	mp-7526	mp-475	wfid_1562345111.58481907
411	PtU	mp-542817	mp-569752	wfid_1562345098.53781532
412	Te5U	mp-651772	mp-28500	wfid_1562345077.40124852
413	U	mp-43	mp-93	wfid_1562345085.52036650
